# Commensal Viruses Promote Intestinal Stem Cell Regeneration Following Radiation Damage by Inhibiting Hyperactivation of RIG‐I and Notch Signals

**DOI:** 10.1002/advs.202505204

**Published:** 2025-07-18

**Authors:** Xiaotong Zhao, Yu Cai, Yujia Hou, Yanjin Wu, Tingting Wei, Lili Li, Zhaojun Duan, Xinran Lu, Jiahui Meng, Haitao Zhou, Qin Wang, Jinhan Wang, Chang Xu, Liqing Du, Saijun Fan, Feng Wang, Qiang Liu, Yang Liu

**Affiliations:** ^1^ State Key Laboratory of Advanced Medical Materials and Devices Tianjin Key Laboratory of Radiation Medicine and Molecular Nuclear Medicine Tianjin Institutes of Health Science Institute of Radiation Medicine Chinese Academy of Medical Sciences & Peking Union Medical College Tianjin 300192 China; ^2^ Department of Genetics School of Basic Medical Sciences Tianjin Medical University Tianjin 300070 China; ^3^ National Institute for Viral Diseases Control and Prevention Chinese Center for Disease Control and Prevention Beijing 102206 China

**Keywords:** fecal virome transplantation, gut commensal viruses, intestinal stem cells, notch signaling pathway, RIG‐I

## Abstract

Radiation‐induced intestinal injury is a common complication of abdominopelvic cancer radiotherapy, often associated with gut bacteriome dysbiosis. However, the involvement of gut virome in this process remains largely underexplored. Here, it was found that radiation disrupted the gut virome, altered the distribution of phages and their bacterial host. Fecal virome transplantation (FVT) from healthy donors ameliorated radiation‐induced intestinal damage and promoted stem cell proliferation by enriching phages targeting *Salmonella*. Conversely, decreased virome load exacerbated intestinal damage, reduced proliferating stem cells, and impaired secretory lineage differentiation. Mechanistically, exacerbated intestinal injury was associated with hyperactivation of RIG‐I and Notch signaling in intestinal stem cells, which was absent in RIG‐I‐deficient mice. Organoids from RIG‐I‐deficient mice displayed decreased Notch signals and increased regenerative capacity post radiation. These findings shed light on the intricate interplay between gut virome, intestinal injury, and stem cell responses, highlighting potential therapeutic interventions for targeting the virome to mitigate radiation‐induced intestinal damage.

## Graphical abstract

Ionizing radiation disrupts gut virome and bacteriome. Gut commensal viruses protect against intestinal damage and promote stem cell regeneration by inhibiting hyperactivation of RIG‐I and Notch signaling in stem cells. Fecal virome transplantation (FVT) from healthy donors can serve as a potential therapeutic intervention by enriching phages targeting pathogenic *Salmonella* of *Proteobacteria* to mitigate radiation‐induced intestinal damage.

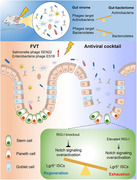

## 1 Introduction

Radiation‐induced intestinal damage is common in patients with abdominopelvic cancer undergoing radiotherapy, limiting their survival and life quality.^[^ ^1^, ^2^ ^]^ This damage often correlates with gut microbial dysbiosis, characterized by changes in bacterial diversity and composition.^[^ ^3^, ^4^, ^5^, ^6^ ^]^ Fecal microbiota transplantation (FMT) from healthy donors effectively alleviates radiation‐induced intestinal injury,^[^ ^7^, ^8^, ^9^ ^]^ but safety concerns persist regarding the transfer of pathogenic microorganisms or toxic compounds.^[^ ^10^, ^11^ ^]^ Except for bacteria, gut viruses (virome) are also one of the main components of gut microbiota, comprising primarily bacteriophages that interact intricately with bacterial hosts.^[^ ^12^, ^13^ ^]^ Although microbiome research has primarily focused on bacteria, the emerging research field of gut virome has gained attention recently. Studies have shown disruptions of the gut virome in intestinal diseases such as Crohn's disease and ulcerative colitis.^[^ ^14^, ^15^ ^]^ Interestingly, antiviral treatment exacerbates colitis in mice,^[^ ^16^ ^]^ while fecal virome transplantation (FVT) without intact bacterial cells has shown promise in alleviating conditions such as recurrent *Clostridioides difficile* infection (CDI),^[^ ^17^ ^]^ necrotizing enterocolitis,^[^ ^18^ ^]^ obesity, and type‐2‐diabetes.^[^ ^19^, ^20^ ^]^ However, the role of gut commensal viruses and the therapeutic potential of FVT in radiation‐induced intestinal injury remain largely unexplored.

Crosstalk between gut commensal viruses and host cells involves conserved pattern recognition receptors (PRRs) that recognize pathogen‐associated molecular patterns (PAMPs) of viral components, such as genomic DNA, single strand RNA (ssRNA), and double strand RNA (dsRNA).^[^ ^21^ ^]^ For instance, TLR3 and TLR7, which detect dsRNA and ssRNA, respectively, can recognize gut commensal viruses to promote IFN‐β secretion by plasmacytoid dendritic cells, offering protection against colitis induced by dextran sulfate sodium (DSS).^[^ ^16^ ^]^ Similarly, the retinoic acid‐inducible gene I (RIG‐I) is also a recognition receptor of gut commensal viral double‐stranded 5′‐triphosphate RNA. RIG‐I signaling in antigen‐presenting cells has been demonstrated to maintain homeostasis of intraepithelial lymphocytes and attenuate intestinal inflammation through the MAVS‐IRF1‐IL‐15 axis.^[^ ^22^ ^]^ Notably, the immune‐modulatory effects of gut commensal viruses may extend to the regulation of intestinal epithelial cells and stem cells, given their pivotal role in maintaining tissue integrity and responding to injury.

Lgr5^+^ intestinal stem cells (ISCs), located at the base of intestinal crypts, play a vital role in maintaining self‐renewal and differentiating into functional intestinal epithelial cells, including secretory lineages (Paneth, goblet, tuft, and enteroendocrine cells) and absorptive lineages (enterocytes).^[^ ^23^ ^]^ Lgr5^+^ ISCs are more sensitive to ionizing radiation and are indispensable in intestinal regeneration after radiation.^[^ ^24^ ^]^ They are precisely regulated by multiple intrinsic and extrinsic factors to maintain stemness and differentiation, such as WNT signaling for self‐renewal and Notch signaling for lineage differentiation.^[^ ^23^, ^24^, ^25^, ^26^, ^27^ ^]^ Moreover, there is growing appreciation for the role of gut microbiota in ISCs regeneration and proliferation. A recent study has shown that germ‐free and antibiotic‐pretreated mice present reduced crypt depth and fewer ISCs, while exposure to FMT from normal mice restores their gut microbes, crypt depth, and ISC numbers.^[^ ^28^ ^]^ Gut bacteria metabolites like valeric acid and lactate have been found to promote ISC regeneration and ISC‐mediated epithelial development.^[^ ^28^, ^29^ ^]^ Furthermore, certain viruses, such as *murine norovirus* infection, can also restore abnormal intestinal crypt structures in germ‐free or antibiotic‐treated mice,^[^ ^30^ ^]^ and *transmissible gastroenteritis virus* infection promotes ISCs self‐renewal through WNT signaling.^[^ ^31^ ^]^ However, the precise roles and mechanisms by which gut commensal viruses influence ISCs regeneration and differentiation, especially in the context of radiation‐induced injury, remain insufficiently understood.

Here, we investigate the role of gut commensal viruses in radiation‐induced intestinal injury and ISCs regeneration. We find that ionizing radiation induces a disruption of the gut virome, whereas FVT prevents intestinal damage and promots stem cell recovery in mice. In contrast, the destruction of gut virome by antiviral treatment can overactivate Notch signals through RIG‐I in ISCs, leading to a decreased number of intestinal epithelial secretory lineage cells and attenuated stem cell regeneration. This study reveals a novel function of gut commensal viruses in radiation‐induced intestinal injury and provides a mechanistic insight into how gut virome regulates ISCs regeneration, offering a potential therapeutic avenue for mitigating radiation‐induced intestinal injury.

## 2 Results

### 2.1 Ionizing Radiation Altered the Composition of Gut Viral and Bacterial Communities

**Figure 1:**
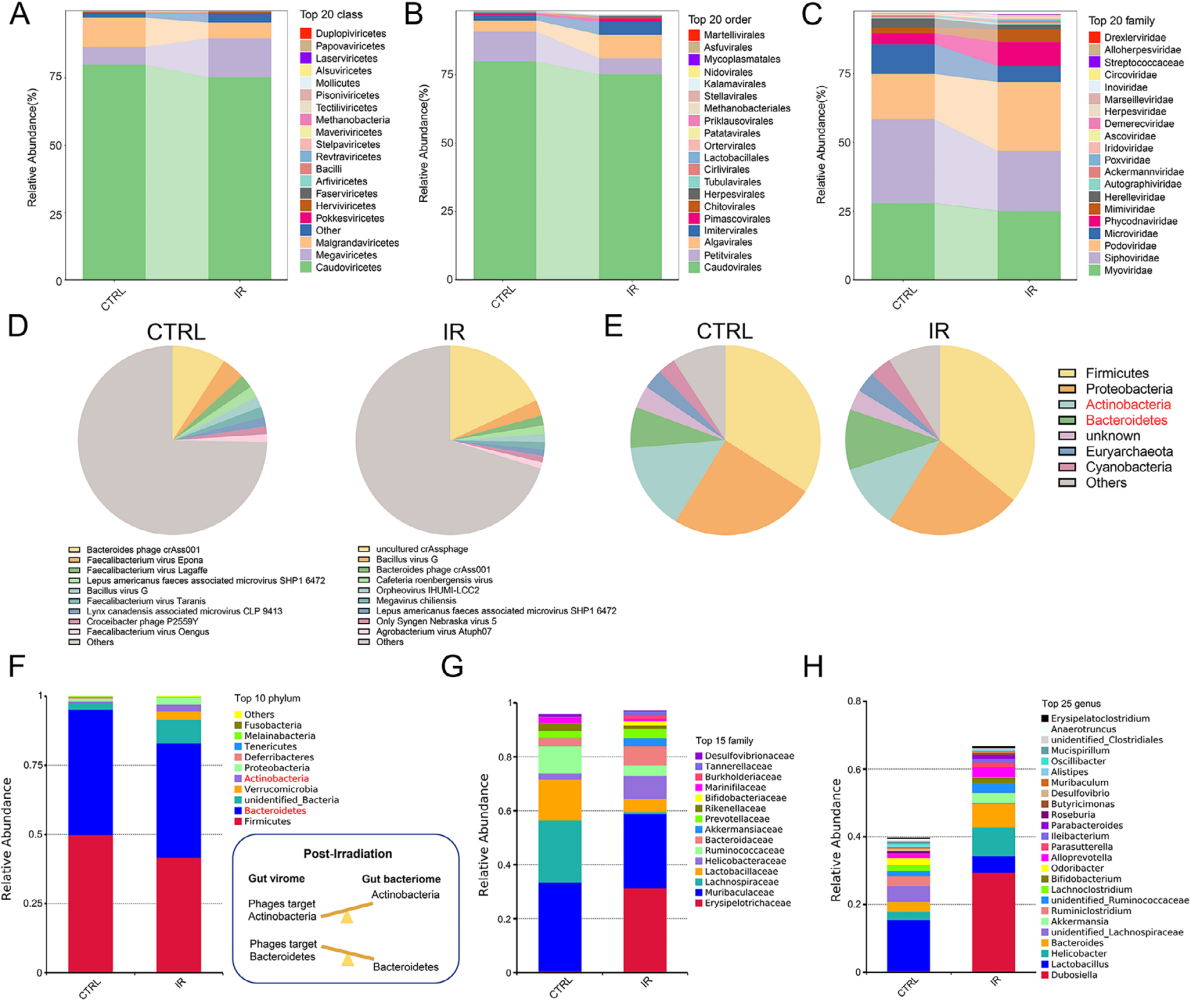
Ionizing radiation altered gut virome and bacteriome composition structure. To analyze the difference in virome, we collected feces from control and 15 Gy irradiated mice and pooled feces from five mice as one sample. A) The relative abundance of viruses at the class level in control and IR‐treated mice was shown. B) The relative abundance of viruses at the order level in control and IR‐treated mice. C) The relative abundance of viruses at the family level in control and IR‐treated mice. D) The proportion of gut virome in the control and IR group at the species level and the top 10 viruses were displayed. E) Distribution of predicted bacterial hosts at the phylum level in control and IR‐treated mice. F) The relative abundance of bacteria at the phylum level in control and IR‐treated mice. G) The relative abundance of bacteria at the family level in control and IR‐treated mice. H) The relative abundance of bacteria at the genus level in control and IR‐treated mice.

To investigate the effects of ionizing radiation (IR) on gut microbiota, we performed sequencing on gut virome and bacteriome. We first isolated the virus‐like particles (VLPs) from mouse feces and conducted metagenomic sequencing on the VLPs. To assess potential bacterial contamination, 16S rRNA quantitative PCR was performed, and the results showed an average reduction of 99.9% in bacterial DNA within the VLP preparations (Figure S1A, Supporting Information). Metagenomic sequencing of fecal VLPs revealed distinct composition of gut virome following ionizing radiation. At the class and order levels, we observed virome shifts in the irradiated mice compared to controls (**Figure** **1**A, B). At the family level, the relative abundance of *Myoviridae*, *Siphoviridae*, and *Microviridae* was decreased in the IR group, while *Podoviridae*, *Phycodnaviridae*, and *Mimiviridae* were more abundant (Figure 1C). Notably, the irradiated group was dominated by *uncultured crAssphage*, which targets the *Bacteroidetes* phylum, accounting for up to 18% of the virome (Figure 1D).

To further explore the host‐phage interactions, we predicted the bacterial hosts of these phages based on VLPs contigs and analyzed these predictions in conjunction with observed alterations in bacterial distribution at the phylum level. The prediction results showed that the IR group had a lower host proportion of *Actinobacteria* and a higher host proportion of *Bacteroidetes* compared to the control group (Figure 1E). Similarly, the results of 16S rRNA gene sequencing of gut bacteriome exhibited that the abundance of *Actinobacteria* was increased in the IR group, and *Bacteroides* was slightly decreased in the IR group when compared with the control group at the phylum level, and these alterations possibly due to the changes of phages (Figure 1F). Additionally, the distinct structure was also observed at the family and genus levels between control and irradiated mice (Figure 1G, H). These findings highlight the significant impact of ionizing radiation on both the gut virome and bacteriome and indicate a dynamic interplay between the virome and bacteriome following radiation exposure.

### 2.2 FVT Attenuated Radiation‐Induced Intestinal Injury by Enriching Phages Targeting *Salmonella*

**Figure 2:**
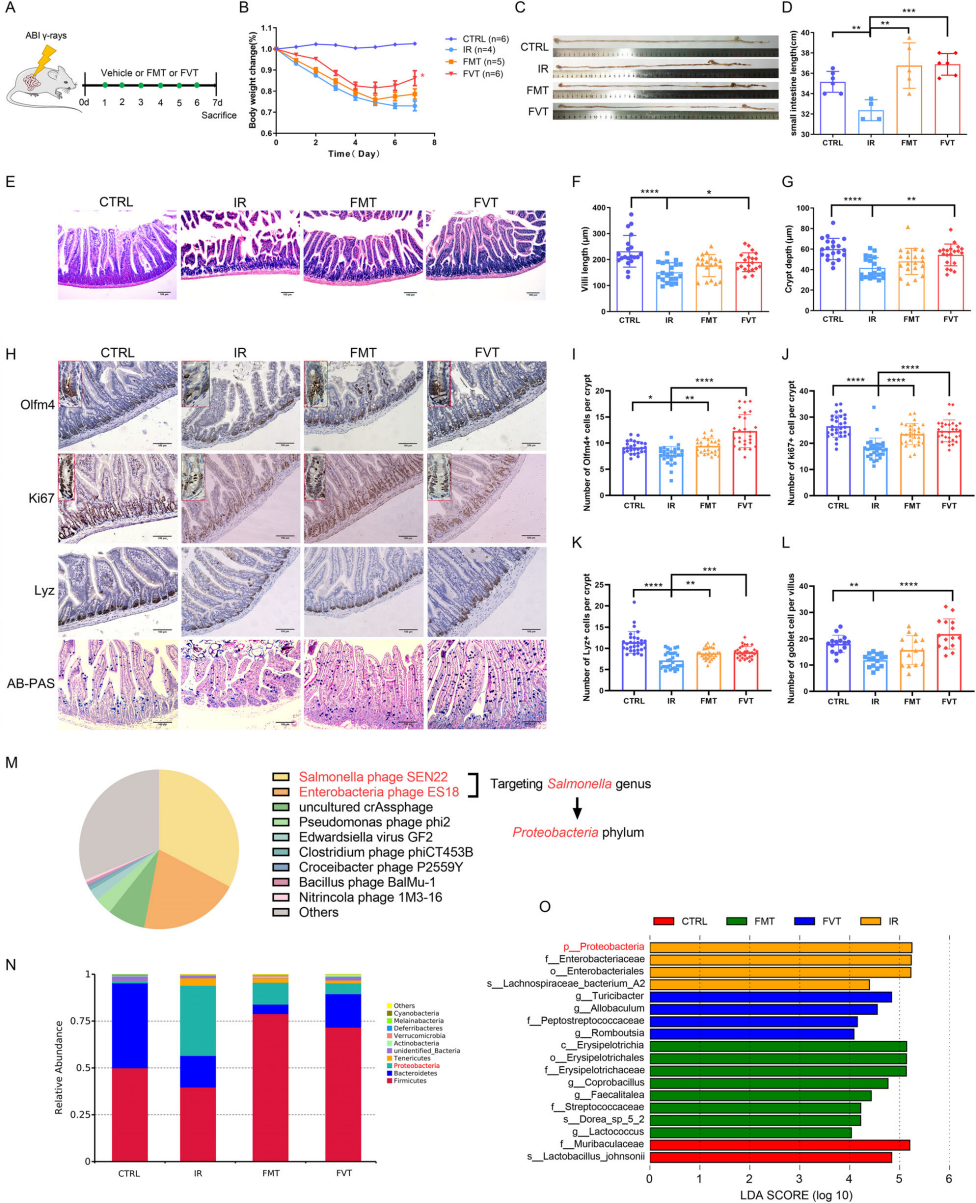
FVT mitigated radiation‐induced intestinal damage and improved ISCs regeneration. A) Schematic diagram of study design. After receiving 18 Gy abdominal irradiation, the mice were transplanted with heat‐inactivated microbes, FMT, and FVT for 6 consecutive days, and euthanized on the seventh day (n = 4–6). B) Body weight changes of mice after different treatments. Representative data was shown from one of two experimental repeats. C,D) The length of the small intestine was measured at 7 days after abdominal radiation. E–G) Representative images of H&E staining and quantitative analysis of villi length and crypt depth at 7 days after IR, FMT, and FVT treatment. Statistical significance was calculated by one‐way ANOVA with Tukey's multiple‐comparisons test. Scale bar = 100 µm. H) Representative images of immunohistochemical staining for stem cell marker Olfm4, proliferating cells marker Ki67, Paneth cells marker Lyz, and AB‐PAS staining for goblet cells. Scale bar = 100 µm. I,J) The number of Olfm4^+^ ISCs and Ki67^+^ proliferating cells in each crypt was measured in different groups. Statistical significance was calculated by one‐way ANOVA with Tukey's multiple‐comparisons test. K,L) Quantitative analysis of the number of Lyz^+^ Paneth cells and goblet cells. Statistical significance was calculated by one‐way ANOVA with Tukey's multiple‐comparisons test. M) The proportion of gut virome in FVT‐treated mice at the species level and the top 10 viruses were displayed. N) The relative abundance of bacteria at the phylum level among mice in four groups. O) The linear discriminant analysis effect size (LEfSe) result represented the significantly different bacteria among the CTRL, IR, FMT, and FVT groups. LDA = 3. Each group contained three independent samples. Data represent the mean ± SEM. ^*^ *P* < 0.05, ^**^ *P* < 0.01, ^***^ *P* < 0.001, ^****^ *P* < 0.0001.

Given that radiation perturbed the gut virome, we explored whether fecal virome transplantation (FVT) could counteract radiation‐induced intestinal injury. To this end, we isolated the microbial and viral components from healthy mice feces and performed heat‐inactivated fecal microbiota transplantation (vehicle), fecal microbiota transplantation (FMT), or fecal virome transplantation (FVT) to mice with 18 Gy abdominal irradiation for 6 consecutive days (**Figure** **2**A). Remarkably, FVT treatment resulted in a significant enrichment of viral counts and minimal 16S rRNA copy numbers (bacterial DNA) compared to controls (Figure S1B, C, Supporting Information). Additionally, we observed that IR caused decreased body weight and shortened small intestine length in mice, both of which were restored by FVT treatment (Figure 2B–D). Further, histologic analysis of the jejunum displayed that the irradiated mice exhibited more severe tissue damage with shortened villi and decreased depth of crypts, while FVT treatment provided more pronounced protection against these injuries compared to FMT treatment (Figure 2E–G). The physiological and histological outcomes underscore the potential benefits of the virome enrichment.

To determine the regeneration capacity of ISCs after ionizing radiation, we assessed their numbers and proliferative potency through immunohistochemical staining for the ISCs marker Olfm4 and the proliferation marker Ki67 (Figure 2H). The number of Olfm4^+^ ISCs in crypts and Ki67‐positive cells was reduced in irradiated mice, but was notably restored following FMT and FVT treatments (Figure 2I, J). Moreover, double immunofluorescence of the Olfm4 and proliferation marker PCNA further confirmed the decreased proliferative potential of stem cells in irradiated mice, while FMT or FVT treatments significantly increased the number of proliferating stem cells (Figure S2, Supporting Information). In addition, we also examined the changes of the secretory lineage cells, including Paneth and goblet cells, which secreted antimicrobial peptides and mucus to maintain intestinal homeostasis (Figure 2H). Lysozyme (Lyz) immunohistochemical staining was performed to detect the number of Paneth cells, and Alcian Blue‐Periodic Acid‐Schiff (AB‐PAS) staining was adopted to evaluate the number of goblet cells. The numbers of Paneth and goblet cells were also decreased after radiation, and were both recovered after FVT treatment (Figure 2K, L). Peripheral blood cell analysis showed lower WBC counts and a disordered blood cell composition in irradiated mice, while FVT increased the WBC counts and restored the distribution of peripheral blood cells to a level more comparable to the control group (Figure S3A–D, Supporting Information). Collectively, these results indicate that FVT from healthy feces can attenuate radiation‐induced intestinal injury, promote stem cell regeneration after injury, and ameliorate hematopoietic damage.

In addition, we also compared the composition of the virome and bacteriome in FVT‐treated mice. The virome in the FVT group was dominated by *Salmonella phage SEN22* and *Enterobacteria phage ES18*, which constituted over 50% of the virome, both targeting *Salmonella* of the *Proteobacteria* phylum (Figure 2 M). Clinical research has identified elevated levels of *Proteobacteria* in patients with radiation enteritis.^[^ ^5^, ^32^, ^33^ ^]^ Similarly, our results of 16S rRNA gene sequencing from mice feces also exhibited the significantly increased abundance of *Proteobacteria* phylum in the IR group, which was reversed by both FMT and FVT treatment. Notably, the FVT group carried a lower abundance of *Proteobacteria* compared to the FMT group (Figure 2N, O). Furthermore, IR decreased the abundance of *Firmicutes*, which was restored following both FMT and FVT treatments, with FMT leading to a more pronounced increase of *Firmicutes* (Figure 2N). The abundance of *Bacteroidetes* was also decreased in the IR group, and further reduced following FMT, whereas no changes were observed in the FVT group compared to the IR group (Figure 2N). Although overall bacterial diversity was not significantly different among the CTRL, IR, FMT, and FVT groups, distinct bacterial species composition was observed (Figure S4A–C, Supporting Information). Moreover, the weighted principal coordinate analysis (PCoA) data revealed distinct clusters of the gut bacteria in FMT or FVT groups compared to the IR group (Figure S4D, Supporting Information). The heatmap of bacteriome at the genus level also exhibited different bacterial compositions among the IR, FMT, and FVT groups (Figure S4E, Supporting Information). At the genus level, FMT treatment significantly increased *Coprobacillus*, *Faecalitalea*, and *Lactococcus*, while FVT treatment significantly increased the abundance of *Turicibacter*, *Allobaculum*, and *Romboutsia* (Figure 2O). Altogether, these findings suggest that FVT modulates the gut bacteriome composition by enriching phages targeting specific bacterial taxa, thereby mitigating radiation‐induced damage.

### 2.3 Gut Commensal Viruses were Required for Protecting the Intestine from Ionizing Radiation

**Figure 3:**
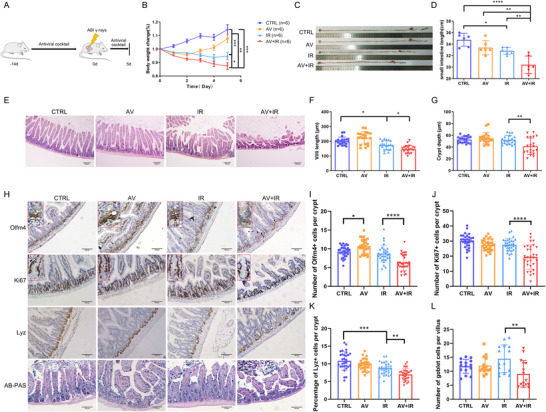
Decreased commensal virus load exacerbated radiation‐induced intestinal damage. A) Schematic diagram of study design. Mice were treated with AV (ribavirin, lamivudine, and acyclovir) for 14 days before 15 Gy abdominal irradiation, and euthanized at day 5 post‐IR (n = 6). B) Body weight changes were monitored for 5 days after radiation. Representative data was shown from one of three experimental repeats. C,D) The length of the small intestine was measured at 5 days after radiation. E) Representative images of H&E‐stained intestine sections. F,G) Quantitative analysis of villi length and crypt depth at day 5 after radiation. Statistical significance was calculated by one‐way ANOVA with Tukey's multiple‐comparisons test. Scale bar = 100 µm. H) Representative immunohistochemistry images of Olfm4 (ISCs), Ki67 (proliferating cells), Lyz (Paneth cells), and AB‐PAS (goblet cells) staining. Scale bar = 100 µm. I–L) Quantitative analysis of the number of positive cells. Statistical significance was calculated by one‐way ANOVA with Tukey's multiple‐comparisons test. Data presented as mean ± SEM. ^*^ *P* < 0.05; ^**^ *P* < 0.01; ^***^ *P* < 0.001; ^****^ *P* < 0.0001.

To further investigate the role of gut commensal viruses in mitigating radiation‐induced intestinal injury and supporting regeneration of ISCs, we treated mice with an antiviral cocktail (AV) for 14 days prior to 15 Gy abdominal radiation (**Figure** **3**A). AV treatment reduced the viral load in the gut (Figure S5A, Supporting Information), and we found that mice in the AV‐treated group (AV+IR) presented more severe tissue damage than those in the AV‐untreated group (IR). The AV‐treated mice exhibited progressive weight loss compared to AV‐untreated mice, indicating a deteriorating condition (Figure 3B). Besides, severe intestinal damage was also displayed by shortened intestine length in AV‐treated mice (Figure 3C,D). Accordingly, AV‐treated mice showed more obvious jejunal histological injury with reduced villi length and crypt depth, compared with AV‐untreated mice (Figure 3E–G). Further, we found that the numbers of Olfm4^+^ ISCs, Ki67^+^ proliferating cells, PCNA^+^ Olfm4^+^ proliferating stem cells, Paneth cells, and goblet cells were all significantly reduced in the AV‐treated group, indicating attenuated ISCs regeneration and reduced secretory lineage cells (Figure 3H–L; Figure S5B, C, Supporting Information). To exclude the direct effect of AV on stem cell regeneration, we isolated small intestinal organoids and treated them with AV and ionizing radiation. The results showed no significant inhibitory effect of AV on organoid growth (Figure S5D–F, Supporting Information). Moreover, AV‐treated mice presented more severe hematopoietic system impairment, as indicated by decreased splenic index, peripheral blood WBC counts, lymphocyte counts (LY), and LY%, along with elevated neutrophil cell percentages (NE%) (Figure S6A–E, Supporting Information). Measurements of cytokine levels in plasma showed that AV treatment decreased the level of anti‐inflammatory cytokines IL‐10 and IL‐5 and increased pro‐inflammatory factor IL‐6, indicating an elevated inflammatory response in AV‐treated irradiated mice (Figure S6F–H, Supporting Information). Collectively, these results further underscore the protective role of gut commensal viruses in mitigating radiation‐induced intestinal injury, promoting ISCs regeneration, and alleviating hematopoietic damage.

### 2.4 Decreased Commensal Virus load Impaired ISCs Regeneration and Increased the *Isg15^+^* ISCs Population in Irradiated Mice

**Figure 4:**
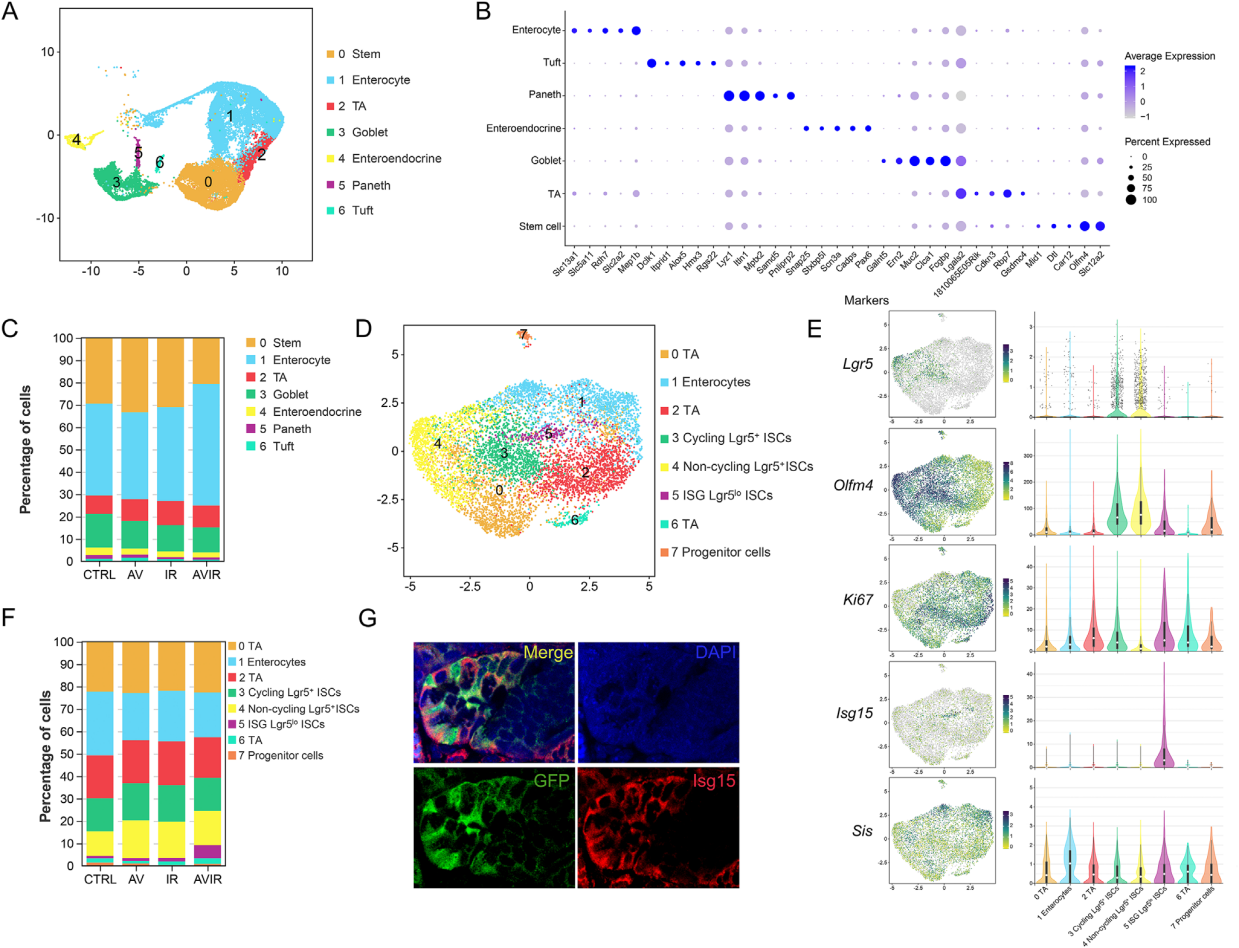
AV cocktail‐treatment decreased stem cells and increased *Isg15* positive ISCs. A) UMAP of 27339 intestinal epithelial cells from control and irradiated mice with or without AV treatment. These cells can be identified as seven epithelial cell types. B) Dot plots revealed the expression of marker genes in seven populations. C) Quantitative results of the percentage of cells per cluster in different mice. D) UMAP of subclustered stem cells. E) UMAP and violin plots illustrated the distribution and expression of marker genes in each stem cell subcluster. F) Quantification analysis of percent cells per stem cell subcluster. G) Representative immunofluorescence images of Isg15 and Lgr5 staining in the intestine of AV‐treated irradiated mice.

To explore the mechanism of commensal viruses in intestinal renewal and differentiation, we isolated intestinal epithelial cells and performed single‐cell RNA sequencing. The results revealed 25 cell clusters comprising intestinal epithelial cells, immune cells, and erythroid cells (Figure S7A,B, Supporting Information). Among epithelial cells, seven distinct clusters were identified based on expression signatures and marker genes (**Figure** **4**A,B). Pseudo‐time analysis revealed that seven epithelial cell types could be divided into three trajectories, differentiated from stem cells and TA cells to secretory lineage and absorptive lineage (Figure S7C, Supporting Information). We further analyzed the proportions of these cell types and found a significant decrease in ISCs and secretory cells, accompanied by an increase in absorptive enterocytes in the AV+IR group (Figure 4C). This indicates that AV treatment inhibites ISCs regeneration and skews intestinal epithelial cell differentiation toward the absorptive lineages.

Given the important role of ISCs in intestinal recovery after injury, we further subclustered the ISCs clusters into eight unique clusters based on transcriptional profiles (Figure 4D). ISCs marker *Lgr5*, *Olfm4*, and proliferative marker *Ki67* exhibited varying distributions across these subclusters (Figure 4E). Based on the different expression profiles of ISCs subclusters, we identified Lgr5^+^ cycling ISCs, Lgr5^+^ non‐cycling ISCs, progenitor cells, TA cells, enterocytes, and a new cell type with high expression of interferon‐stimulated gene 15 (*Isg15*) and low expression of *Lgr5*, which we termed “ISG Lgr5^lo^ ISCs” (Figure 4D, E; Figure S7D, Supporting Information). Interestingly, both the Lgr5^+^ cycling and non‐cycling ISCs showed a slight decrease in the AV+IR group, while the new cell type of ISG Lgr5^lo^ ISCs significantly increased following AV treatment (Figure 4F). Immunofluorescence results also confirmed that a portion of Lgr5^+^ ISCs expressed Isg15 in the intestine of AV‐treated irradiated mice (Figure 4G).

**Figure 5:**
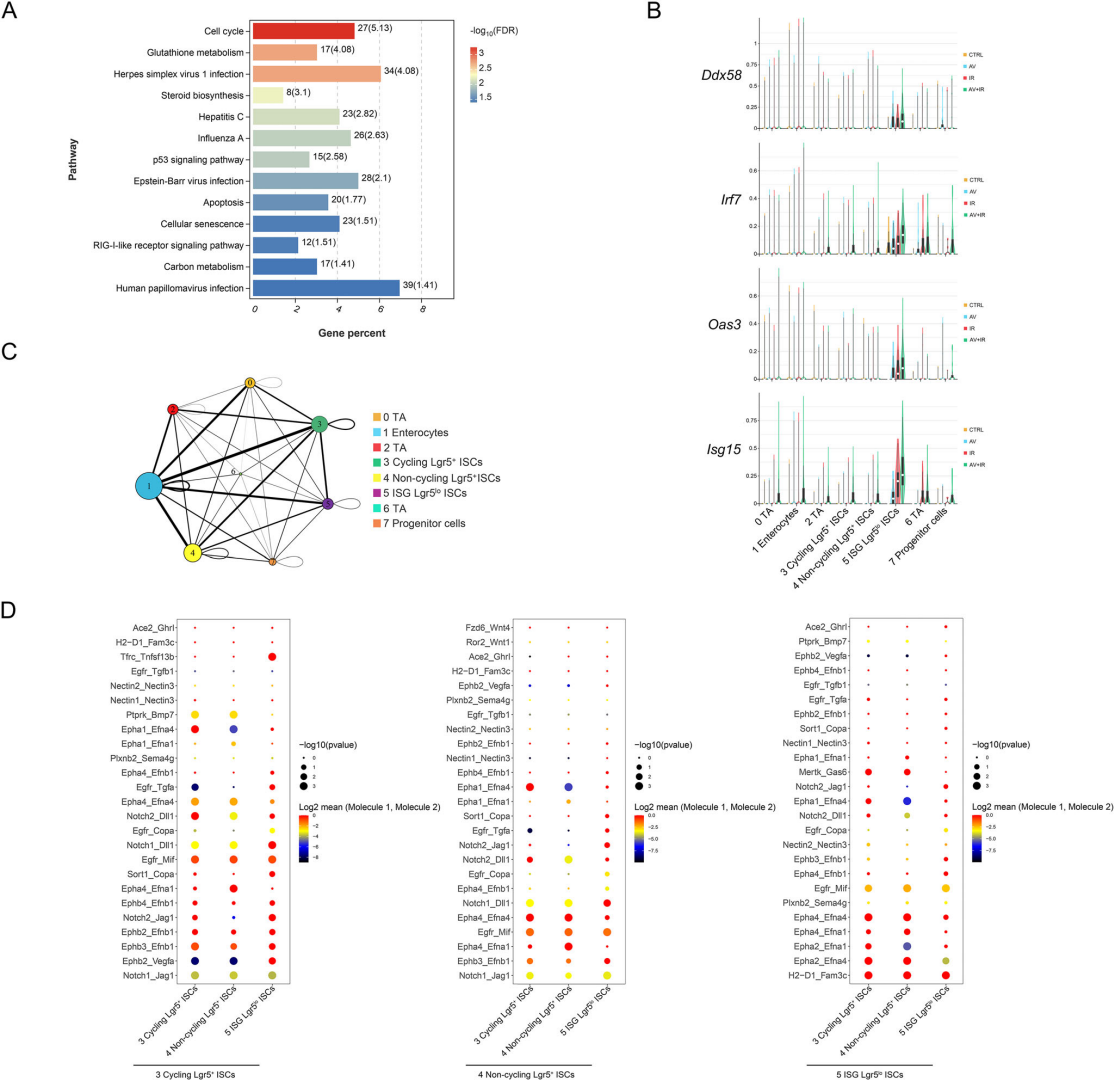
Functional enrichment of ISG Lgr5^lo^ ISCs subcluster and potent ligand‐receptor interactions with other subclusters. A) KEGG functional enrichment analysis of upregulated genes in the ISG Lgr5^lo^ ISCs cluster. B) Violin plots of representative genes of the RIG‐I‐like receptor signaling pathway. C) Cell interaction network among all stem cell subclusters. Bubbles represent cellular subpopulations, and the size of bubbles was determined by the number of significantly enriched ligand‐receptor pairs between a subpopulation and all of its interacting subpopulations. The line represented the number of significantly enriched ligand‐receptor pairs between subpopulations, and the thicker the line indicated that the more significantly enriched ligand‐receptor pairs between subpopulations, the stronger the communication relationship between subpopulations. D) The bubble plot depicted significant interactions between ISG Lgr5^lo^ ISCs and Lgr5^+^ cycling or non‐cycling cells.

Activation of ISGs typically occurs through PRRs triggered by PAMPs such as viral nucleic acids.^[^ ^21^ ^]^ To further determine the transcriptional changes of ISG Lgr5^lo^ ISCs, we performed KEGG and GO functional enrichment analysis of upregulated genes. The RIG‐I‐like receptor signaling pathway, which is crucial in virus‐host interactions, was enriched in the ISG Lgr5^lo^ ISCs (**Figure** **5**A). Besides, many RNA metabolic process‐related functions were also enriched in the ISG Lgr5^lo^ ISCs, which indicated the underlying mechanism of RIG‐I signals’ activation (Figure S7E, Supporting Information). In addition, the ISG Lgr5^lo^ ISCs present transcriptomic features indicative of regenerative capacity, consistent with the elevated proportion of this subcluster (Figure S7F, Supporting Information). Further, we analyzed the expression of genes associated with the RIG‐I‐like receptor signaling pathway, and the results showed that these genes were significantly elevated in the ISG Lgr5^lo^ ISCs of AV‐treated irradiated mice (Figure 5B). To explain the relationship among ISG Lgr5^lo^ ISCs and other subclusters in stem cells, we performed ligand‐receptor interactions analyses using CellPhoneDB to predict the enriched interactions between two cell types. Strong ligand‐receptor interactions were found between ISG Lgr5^lo^ ISCs and cycling Lgr5^+^ ISCs, non‐cycling Lgr5^+^ ISCs, and enterocytes, respectively (Figure 5C). Notably, the Notch signaling pathway, which is closely related to ISCs functions, was significantly enriched in ligand‐receptor interactions among the ISG Lgr5^lo^ ISCs, cycling and non‐cycling Lgr5^+^ ISCs clusters (Figure 5D). Overall, these findings suggest that the reduction of intestinal commensal virus load decreases the number of cycling and non‐cycling ISCs, increases heterogeneity of ISCs, potentially influences RIG‐I and Notch signaling in ISCs, ultimately impairing intestinal regeneration.

### 2.5 The RIG‐I Signaling was Essential for Exacerbating Intestinal Damage when the Commensal Virus Load was Reduced

**Figure 6:**
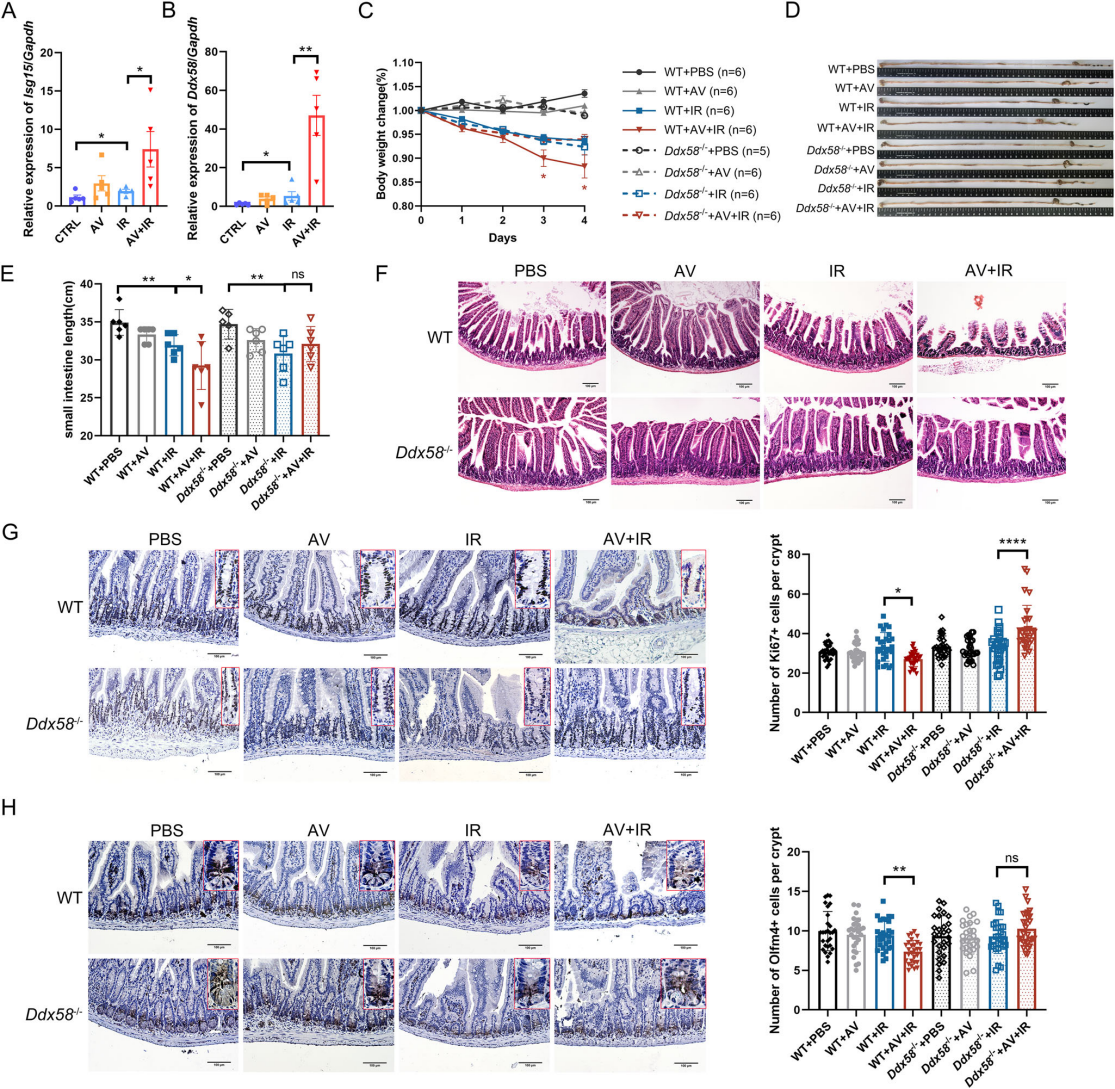
AV cocktail relied on RIG‐I regulating stem cells and participating in intestinal damage. A,B) The expression of *Isg15* and *Ddx58* in stem cells. Each dot represented one mouse (n = 5). C) The results of body weight changes of WT and *Ddx58* ^−/−^ mice with or without AV and ionizing radiation treatment (n = 5‐6). D,E) The representative images of the small intestine and quantitative analysis of the intestine length of WT and *Ddx58* ^−/−^ mice with or without AV and ionizing radiation treatment. F) Representative images of H&E staining of the intestine in different mice. G,H) Representative images and quantitative analysis of immunohistochemical staining for Ki67 and Olfm4. Statistical significance was calculated by one‐way ANOVA with Tukey's multiple‐comparisons test. Data presented as mean ± SEM. ^*^ *P* < 0.05; ^**^ *P* < 0.01; ^***^ *P* < 0.001; ^****^ *P* < 0.0001.

To further investigate how the AV cocktail reduced the proportion of ISCs, we sorted Lgr5^+^ ISCs using *Lgr5* ^EGFP‐IRES‐CreERT2^ mice, and examined the levels of *Isg15* and RIG‐I (*Ddx58*) (Figure S8A, Supporting Information; **Figure** **6**A,B). The results of ISCs showed an increase level of *Isg15* and *Ddx58* after IR, which were further elevated in AV‐treated irradiated mice (Figure 6A,B). Besides, AV‐treatment significantly increased the expression of genes associated with the RIG‐I‐like receptor signaling pathway, including *Mavs*, *Irf3*, *Irf7*, *Ifnb1*, and *Stat1* in Lgr5^+^ ISCs compared to the IR group (Figure S8B–F, Supporting Information). Interestingly, AV treatment did not significantly affect *Isg15* and *Ddx58* expression in the broader intestinal tissue, indicating that these changes were specific to ISCs. (Figure S8G,H, Supporting Information). Furthermore, we explored the significance of RIG‐I signaling in exacerbated intestinal damage due to reduced commensal virus load using RIG‐I‐deficient (*Ddx58* ^−/−^) mice. Our observations in RIG‐I‐deficient mice showed that the AV cocktail no longer led to reductions in body weight or intestinal length compared to irradiated wild‐type (WT) controls (Figure 6C–E). Histological analysis revealed severe intestinal damage in AV‐treated WT irradiated mice, while *Ddx58* ^−/−^ mice displayed no significant damage (Figure 6F). Moreover, in wild‐type mice subjected to AV and ionizing radiation, there was a considerable decrease in the number of Ki67^+^ cells, but in *Ddx58* ^−/−^ mice, the number of proliferating cells increased with AV treatment (Figure 6G). Likewise, while the number of Olfm4‐positive stem cells significantly decreased in AV and irradiation‐treated wild‐type mice, there was no significant change observed in *Ddx58* ^−/−^ mice (Figure 6H). Similarly, the number of PCNA^+^ Olfm4^+^ proliferating stem cells was significantly reduced in AV‐treated wild‐type mice after radiation, whereas no significant change was observed in AV‐treated *Ddx58* ^−/−^ mice under the same conditions (Figure S9, Supporting Information). In addition, the results of peripheral blood cell analysis showed significantly decreased WBC, LY, RBC, and HGB in the AV‐treated wild‐type mice after radiation, but no significant changes were observed in the AV‐treated *Ddx58* ^−/−^ mice after radiation (Figure S10, Supporting Information). Thus, radiation and AV cocktail enhanced RIG‐I signals in ISCs, highlighting the dependence of gut commensal viruses on RIG‐I to modulate radiation‐induced intestinal damage and ISCs regeneration.

### 2.6 Viral Load Reduction Hyperactivated the Notch Signaling Pathway in ISCs Through RIG‐I

**Figure 7:**
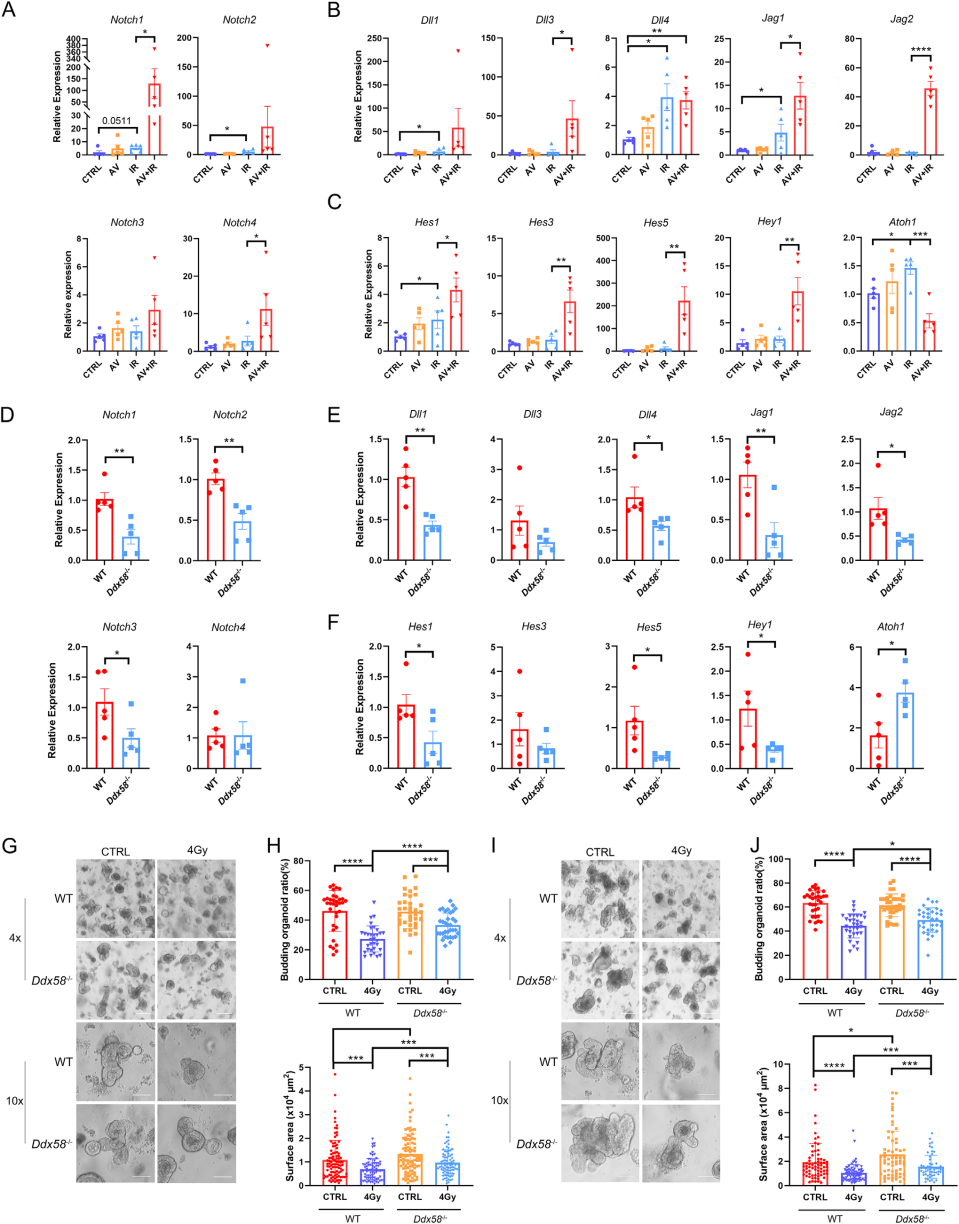
Decreased viral load activated the Notch signaling through RIG‐I. A) The expression of Notch receptors in Lgr5^+^ stem cells after AV treatment and radiation. Each dot represented one mouse (n = 5). B) The expression of Notch ligands in Lgr5^+^ stem cells after AV treatment and radiation. Each dot represented one mouse (n = 5). C) The expression of Notch target genes and *Atoh1* in Lgr5^+^ stem cells after AV treatment and radiation. Each dot represented one mouse (n = 5). D–F) The expression of Notch receptors, ligands, target genes, and *Atoh1* in WT and *Ddx58* ^−/−^ intestinal crypt organoids. Data from five independent replicates. G,H) Representative images and quantitative analysis of organoid formation of crypts obtained from WT and *Ddx58* ^−/−^ mice at 24 h after radiation. Data from three independent replicates. I,J) Representative images and quantitative analysis of organoid formation of crypts obtained from WT and *Ddx58* ^−/−^ mice at 48 h after irradiation. Data from three independent replicates. Data presented as mean ± SEM. ^*^ *P* < 0.05; ^**^ *P* < 0.01; ^***^ *P* < 0.001; ^****^ *P* < 0.0001.

We further evaluated the gene expression of Notch signaling in ISCs. The results revealed that IR led to a modest increase in certain Notch signaling genes in stem cells, whereas AV treatment significantly elevated the levels of the majority of genes of Notch signaling (**Figure** **7**A–C). Decreased expression of Atoh1 is typically associated with reduced differentiation into secretory lineage cells,^[^ ^34^ ^]^ and AV treatment decreased *Atoh1* level in irradiated stem cells (Figure 7C). This observation is consistent with results from immunohistochemistry and single‐cell RNA sequencing, both of which indicated a reduction in secretory lineage cells in AV‐treated irradiated mice. The regeneration of ISCs is regulated by a combination of endogenous and exogenous factors, including dietary factors, gut microbes, immune microenvironment, and intracellular factors etc. Intestinal crypt organoids are an ideal system for studying the function of ISCs in vitro. They contain ISCs that can differentiate into different types of intestinal epithelial cells, and provide a controlled environment that allows for the functional assessment of stem cells independent of systemic influences, such as microbial or immune‐derived signals.^[^ ^35^ ^]^ Therefore, to further clarify the direct effects of RIG‐I on Notch signaling to promote stem cells regeneration, and eliminate the indirect influence of gut microbes and immune cells, we isolated and cultured the intestinal crypt organoids from WT and *Ddx58* ^−/−^ mice. The expression of Notch receptors (*Notch1*, *Notch2*, *Notch3*), ligands (*Dll1*, *Dll4*, *Jag1*, *Jag2*) and their target genes (*Hes1*, *Hes5*, *Hey1)* was significantly decreased, while *Atoh1* was markedly increased in *Ddx58* ^−/−^ organoids (Figure 7D–F). Meanwhile, small intestinal crypts isolated from *Ddx58* ^−/−^ mice also exhibited diminished Notch signals regardless of radiation exposure, in comparison with wild‐type mice (Figure S11, Supporting Information). In addition, we performed radiation to WT and *Ddx58* ^−/−^ organoids and observed that *Ddx58* ^−/−^ organoids exhibited higher germination rates and areas compared to wild‐type organoids at 24 and 48 h after radiation (Figure 7G–J). Altogether, these findings provide additional support for the role of RIG‐I in modulating Notch signaling to regulate stem cell regeneration.

## 3 Discussion

Increasing evidence has suggested that gut microbiota, especially commensal bacteria, play a role in radiation‐induced intestinal damage.^[^ ^3^ ^]^ Here, we demonstrated that gut commensal viruses also have a protective role in the development of radiation‐induced intestinal damage. We found that radiation caused the disorder of gut virome, while FVT from healthy mice could mitigate radiation‐induced intestinal damage and promote ISCs regeneration via enriching phages targeting *Salmonella*. Conversely, decreased load of gut commensal viruses made mice more vulnerable to ionizing radiation. Mechanistically, we found that the gut virome is involved in radiation‐induced damage by altering the ISCs population, mainly through its impact on RIG‐I and Notch signaling pathways. The reduced viral load led to excessive activation of RIG‐I and Notch signaling in ISCs, while RIG‐I deficiency exhibited lower Notch signals and improved organoid formation after radiation. These findings suggest that gut commensal viruses facilitate ISCs regeneration after injury by inhibiting excessive RIG‐I activation, and thus reducing Notch signaling over‐activation in stem cells, ultimately providing intestinal radioprotection.

Intestinal homeostasis is regulated by a combination of factors, including genetic factors, the immune system, and gut microbiota.^[^ ^36^ ^]^ Gut microbiota is a diverse community comprising bacteria, fungi, and viruses, with research on gut commensal viruses only gaining significant attention in recent years. Gut virome encompasses eukaryotic viruses and bacteriophages, dominated by phages.^[^ ^13^ ^]^ Mounting evidence has connected dysbiosis in the gut virome to various disease pathologies. For instance, in CDI, there was an increased abundance of *Caudovirales* and *Anelloviridae*, alongside a decrease of *Microviridae*.^[^ ^37^ ^]^ Similarly, the relative abundance of *Caudovirales* was increased in inflammatory bowel disease (IBD), along with a decrease of *Microviridae*.^[^ ^38^ ^]^ In our study, we also found the dysregulation of gut virome in radiation‐induced intestinal damage, characterized by decreased relative abundance of *Myoviridae*, *Siphoviridae*, *Microviridae*, and an increase of *Podoviridae*, *Phycodnaviridae*, and *Mimiviridae* at the family level. In addition, the administration of an antiviral cocktail further exacerbated radiation‐induced intestinal damage, whereas FVT effectively restored it. This aligns with findings in other diseases, where FVT, as a new therapeutic approach for regulating gut microbiota homeostasis, has shown efficacy in alleviating CDI, necrotizing enterocolitis, and type 2 diabetes.^[^ ^17^, ^18^, ^20^ ^]^

Fecal microbiota transplantation (FMT) is an effective therapeutic strategy for restoring gut microbiota homeostasis, particularly in the treatment of CDI and other dysbiosis‐related diseases, by transferring diverse microbial communities.^[^ ^39^, ^40^, ^41^ ^]^ However, FMT has high heterogeneity due to variations in dosage and delivery routes, along with safety concerns of transferring pathogenic bacteria and adverse reactions in some patients.^[^ ^42^ ^]^ In response to these challenges, a shift from FMT to transplantation of defined fecal microbial fractions (bacteria and viruses) has emerged. Washed microbiota transplantation (WMT) selectively removes unwanted components and is delivered via colonic transendoscopic enteral tubing, reducing adverse reactions and improving treatment precision, with benefits seen in conditions like ulcerative colitis and radiation‐induced enteritis.^[^ ^9^, ^42^, ^43^ ^]^ Gut commensal virome is the second most abundant species in the gut microbiota, and fecal virome transplantation (FVT) is another promising alternative, which focuses on transplanting the viral component of donor feces. By excluding bacterial components, FVT minimizes the risk of transmitting pathogenic bacteria while preserving the modulatory capacity of the virome. Besides, the main component of FVT is bacteriophages, which can be stably colonized in the recipient following transplantation and are better correlated with successful FMT than bacterial communities.^[^ ^44^ ^]^ Furthermore, bacteriophages play an important role in maintaining the integrity of the intestinal epithelial barrier, regulating intestinal immunity, and shaping the community structure and stability of the human intestinal microbiota.^[^ ^45^, ^46^, ^47^, ^48^ ^]^

Bacteriophages, which use bacteria as hosts, exhibit diverse reproductive strategies. Lytic phages can directly affect gut bacterial abundance, diversity, and metabolism by lysing bacteria.^[^ ^49^, ^50^ ^]^ Conversely, lysogenic phages do not lysate bacteria but insert their genomes into the genome of the host bacteria.^[^ ^49^ ^]^ In our study, the distribution of *uncultured crAssphage*, which is a virulent phage targeting the *Bacteroidetes* phylum,^[^ ^51^ ^]^ was most abundant in the IR group. Prediction of bacterial host from phage VLPs showed an increase in the proportion of *Bacteroidetes* and a decrease in the proportion of *Actinobacteria* in the IR group, which were contrary to the results of 16S rRNA gene sequencing. This indicates that gut virome can influence bacterial communities and thus contribute to radiation‐induced damage. Further analyzing the core virome of the FVT group, we found *Salmonella phage SEN22* and *Enterobacteria phage ES18*, which target the *Proteobacteria* phylum pathogenic *Salmonella* genus, were together accounted for more than 50% of the total virome. This aligns with the observed reduction of *Proteobacteria* abundance in the FVT group. Remarkably, the abundance of *Proteobacteria* was elevated in the gut microbiota of patients with radiation enteritis.^[^ ^5^, ^32^, ^33^ ^]^ *Proteobacteria* includes many pathogenic species,^[^ ^52^ ^]^ hence expansion of *Proteobacteria* may contribute to the pathogenesis of radiation enteritis, and FVT mitigates radiation‐induced intestinal injury by enriching phages targeted pathogenic bacteria and regulating the relative abundance and functionality of bacterial hosts.

ISCs are the source of all intestinal epithelial cell types. When intestinal injury occurs, ISCs proliferate rapidly to fill the damaged crypts and intestinal epithelial cells, while when ISCs are damaged, the missing cells cannot be replenished in time, resulting in compromised intestinal integrity.^[^ ^53^, ^54^ ^]^ Therefore, the regenerative capacity of ISCs after injury is crucial for effective intestinal repair. Previous studies have highlighted the role of gut bacteria and probiotics in regulating ISC self‐renewal and regeneration through various mechanisms.^[^ ^55^ ^]^ In this study, we shed light on another important component, gut commensal viruses, which also contribute to promoting ISCs regeneration, specifically through a mechanism involving RIG‐I inhibition. RIG‐I was predominantly expressed in villi along the crypt/villus axis,^[^ ^56^ ^]^ and RIG‐I agonist 3pRNA can reduce the damage of the small intestine when administered before or concurrently with allo‐HSCT (allogeneic hematopoietic stem cell transplantation). However, this protective effect is lost if the agonist is administered after allo‐HSCT.^[^ ^57^ ^]^ Thus, the precise role of RIG‐I in intestinal injury remains elusive. In this study, we focused on the interaction between RIG‐I and ISCs. Our results exhibited significant overactivation of RIG‐I in ISCs of AV‐treated irradiated mice, and AV treatment did not exacerbate intestinal injury in mice lacking RIG‐I, which suggests a more intricate regulatory role for RIG‐I in the regeneration of intestinal stem cells.

Although DNA viruses comprise the majority of the gut virome, activation of the dsRNA‐recognizing receptor RIG‐I by disordered virome is still plausible. RNA viruses are more unstable; hence, they are potentially underrepresented in metagenomic sequencing.^[^ ^58^ ^]^ In addition, DNA viruses produce RNA intermediates during replication, which can be recognized by the dsRNA‐recognizing receptors.^[^ ^59^ ^]^ On the other hand, viral infection can cause host RNA dysregulation, while host RNAs such as vault RNAs, mitochondrial RNAs, circular RNAs, and certain noncoding RNAs (ncRNAs) have been confirmed to activate RIG‐I.^[^ ^60^, ^61^, ^62^ ^]^ In our results, we found RNA processes and ncRNA metabolic processes were significantly enriched in ISG Lgr5^lo^ ISCs via GO function enrichment analysis, so this could be another reason why RIG‐I was over‐activated.

Notch signaling is a conserved signaling pathway that plays a central role in cell differentiation, proliferation, and apoptosis as well as in the development of multiple tissues. In the context of the immune response during viral infections, there is evidence of crosstalk between the Notch signaling and RIG‐I pathway. For instance, studies have shown that dengue virus (DENV) infection can lead to an increase in Notch ligands and receptors in certain immune cells, while silencing PRR molecules such as TLR3 and RIG‐I reduced DENV‐induced Notch ligand expression levels.^[^ ^63^ ^]^ Similarly, in influenza A virus‐infected macrophages, there was an increase in expression of *Dll1*, a Notch ligand, in a RIG‐I‐induced IFN‐I‐dependent manner.^[^ ^64^ ^]^ Our study extends these findings by demonstrating that RIG‐I can also modulate Notch signaling in ISCs. Notch signaling is indispensable for ISCs regeneration after injury, and its inhibition is usually associated with reduced ISCs proliferation.^[^ ^27^ ^]^ However, recent reports showed that the deletion of cellular communication network factor 1 (CCN1) led to decreased Notch signaling, but increased stem cell expansion and organoid formation.^[^ ^65^ ^]^ In our study, RIG‐I knockout organoids exhibited both reduced Notch signaling and increased organoid formation. Additionally, our study revealed an increase in Notch signaling after radiation, which was further elevated in ISCs from the AV+IR group, accompanied by a decrease ISCs in the AV+IR group. This suggests that excessive Notch activation in ISCs may have detrimental effects, and the detailed mechanisms require further exploration. Importantly, hyperactivation of Notch signaling is known to suppress ISC differentiation into secretory lineages.^[^ ^66^ ^]^ Consistent with this, the AV+IR group, with reduced viral load, exhibited significantly fewer secretory cells, whereas the FVT group, with a supplementation of normal gut virome, showed an increase in secretory cells. These results underscore the role of gut commensal viruses in mitigating excessive Notch activation by regulating RIG‐I, thereby maintaining functional homeostasis of ISCs after injury.

In addition to the protective effect against intestinal damage, we also observed an improvement in radiation‐induced hematopoietic damage by gut commensal viruses. The results showed that FVT ameliorated hematopoietic damage, while AV treatment exacerbated hematopoiesis in wild‐type mice. Notably, these effects were abolished in *Ddx58* ^−/−^ mice, suggesting that RIG‐I is a critical mediator in this process. RIG‐I has been shown to play an important role in the development and functional maintenance of the hematopoietic system. For instance, it has been reported that RIG‐I or MDA5 deficiency cannot initiate primitive hematopoietic precursors and disrupts the emergence of myeloid and erythroid cell lineages in zebrafish.^[^ ^67^, ^68^ ^]^ Conversely, in human CD34^+^ cells, activation of RIG‐I/MDA5 by poly I: C promotes apoptosis of human hematopoietic stem cells.^[^ ^69^ ^]^ Besides, excessive expression of RIG‐I disrupted the clonogenicity and bone‐forming ability of BMSCs, and particularly their function of supporting hematopoietic stem cell expansion in vitro and engraftment in vivo.^[^ ^70^ ^]^ In addition, RIG‐I has also been implicated in inhibiting leukemia cell proliferation and leukemia stem cell maintenance through STAT1 activation and downstream AKT regulation.^[^ ^71^ ^]^ However, the precise mechanisms by which gut virome‐mediated RIG‐I activation modulates hematopoietic homeostasis in the context of radiation injury remains to be elucidated and warrants further investigation.

In conclusion, our study emphasizes the critical role of gut commensal viruses in maintaining intestinal homeostasis and fostering ISCs regeneration, and uncovers the role of RIG‐I in intestinal stem cells, specifically in regulating Notch signaling and stem cell differentiation in response to commensal virus perturbation. Furthermore, FVT emerges as a promising intervention to mitigate radiation‐induced intestinal damage, offering novel avenues for preventive and therapeutic approaches against such injuries.

## 4 Experimental Section

### Mice and Antiviral Cocktail Treatment

*Lgr5* ^EGFP‐IRES‐creERT2^ mice were provided by Dr. Haowen Zhang (Soochow University). RIG‐I‐deficient (*Ddx58* ^−/−^) mice were offspring of heterozygotes, which were purchased from Shanghai Model Organisms Center, Inc. All animal experiments were performed in mice with a C57BL/6J background, and mice aged four to eight weeks old, sex matched, were used in all assays. After arriving in the new environment, the mice were first kept in large cages for a week, and cage cleaning was kept to once per week, and then randomly assigned to different treatment groups. During the experiments, the cages were additionally kept clean and prevent microbe‐filled fecal pellets from being ingested. All mice were kept in a specific pathogen‐free environment with 22 ± 2 °C, 40–70% humidity, and maintained 12 h day‐night cycle in accordance with the National Research Council's Guidelines for the Care and Use of Laboratory Animals. To decrease the viral load, mice were divided into four groups, CTRL and IR group gavage with saline for 14 days, while AV and AV+IR group were administrated with antiviral cocktail (AV, 30 mg/kg/day ribavirin, 10 mg/kg/day lamivudine, and 20 mg/kg/day acyclovir) for 14 days. Mice in the IR and AV+IR groups received 15 Gy abdominal irradiation. All mice received saline or AV for 4 days, and were euthanized on the fifth day after irradiation. Every mouse was considered to be an experimental unit.

### Ionizing Radiation

Ionizing radiation was performed using a Gammacell‐40 ^137^Cesium gray irradiator (Atomic Energy of Canada Ltd.). Mice were anesthetized and subjected to localized abdominal irradiation at a dose rate of 0.85 Gy min^−1^ with 3 cm diameter circular radiation field.

### Preparation of Donor Microbiota and Virome

Fresh feces from normal wild‐type C57BL/6J mice were immediately placed on ice and dissolved in sterile saline to configure a 10% suspension. The suspension underwent centrifugation at 800 rpm for 5 min to remove fecal debris and host cells. The supernatant was divided into three parts, one part was heated at 100 °C for 30 min to inactivate live microorganisms as vehicle reagent and administered by gavage to irradiated (IR) mice, one part was kept on ice for the fecal microbiota transplantation (FMT), and the last part was filtered through a 0.45 µm filter (Millipore) and the filtrate was used as the fecal virome transplantation (FVT). The different components were fed to the irradiated mice by gavage for six consecutive days, and the mice were euthanized on the seventh day.

### Virome Isolation and Metagenomic Sequencing

For virome sequencing, feces from five mice were pooled as one sample. Feces were collected, snap‐frozen in liquid nitrogen, and stored at −80 °C for subsequent sequencing and analysis. The isolation and enrichment of the virome were performed according to methods previously reported.^[^ ^22^, ^72^ ^]^ Briefly, feces were swirled and dissolved with sterilized SM buffer (1 M Tris‐HCl (pH7.5), 1 M NaCl, 800 mM MgSO_4_) into a 10% suspension, and then centrifuged at 2500 g for 10 min at 4 °C. The supernatant was sequentially filtered through 0.45 µm (Millipore) and 0.22 µm filters (Millipore) to remove cell debris and bacteria. The filtrate was concentrated using a 100 kDa ultrafiltration tube (Millipore) and centrifuged at 5000 g for 40 min at 4 °C to concentrate the virus‐like particles (VLPs). The concentrated VLPs were treated with DNase I (Sigma Aldrich) and RNase A (Roche) at 37 °C for 1 h to remove free DNA and RNA. Viral RNA was extracted using the QIAamp Viral RNA Mini Kit (Qiagen), and double‐stranded cDNA was synthesized using the PrimeScript Double‐Strand cDNA Synthesis Kit (Takara) according to the manufacturer's instructions. Subsequently, Illumina TruSeg DNA library (Illumina) was prepared and sequenced on Illumina NovaSeq/HiSeq platform using 2 × 150‐base pair paired‐end sequencing. After quality control, the raw data were spliced and annotated for subsequent analysis. The hosts of phages were predicted by CHERRY, the latest computational method, with high accuracy.^[^ ^73^ ^]^

### 16S Ribosomal RNA Gene Sequencing

DNA extraction from feces was performed using TIANamp Stool DNA Kit (Tiangen) according to the provided instructions, followed by PCR amplification using universal V4 region primers. The primer sequences used were 515F, GTGCCAGCMGCCGCGGTAA, and 806R, GGACTACHVGGGTWTCTAAT. After purification of the PCR products, they were used to construct the DNA library using the Ion Plus Fragment Library Kit 48 rxns kit (Thermo Fisher Scientific), and then sequenced on the Ion S5TMXL platform. The raw data were subjected to quality control and screening, with low‐quality and chimeric sequences removed using Cutadapt (V1.9.1 http://cutadapt.readthedocs.io/en/stable/) and Vsearch (https://github.com/torognes/vsearch/). Valid sequences were then clustered and annotated for analysis.

### Isolation of Intestinal Epithelial Cells

Intestinal epithelial cells were isolated using previously described methods.^[^ ^74^ ^]^ Fresh small intestines were washed with PBS and dissected longitudinally. The tissue was further washed with PBS to remove fecal debris and subsequently cut into ≈5 mm pieces. The tissue pieces were treated with 30 mm EDTA and 1.5 mM DTT on ice for 20 min. Subsequently, they were transferred into a 15 mL centrifuge tube containing RPMI medium (Hyclone) with 30 mM EDTA for digestion at 37 °C for 8 min. After shaking for 30 s, the supernatant was taken to a new centrifuge tube and centrifuged at 1000 g for 5 min at 4 °C. The precipitate was resuspended in RPMI medium containing collagenase II (1 mg mL^−1^) (Gbico), collagenase IV (1 mg mL^−1^) (Gbico), and trypsin (Solarbio), and then digested at 37 °C for 10 min. Following digestion, FBS (Gbico) and DNase I (Sigma–Aldrich) were added to halt the process. Single cells were filtered through a 40 µm strainer, followed by centrifuging. The final single cells were assayed for viability and counted after medium resuspension.

### Single‐Cell RNA Sequencing and Analysis

In single‐cell sequencing experiments, epithelial cells from three mice were combined and treated as one sample. Single cell was collected to prepare libraries using Chromium Next GEM Single Cell 3′ Reagent Kits v3.1 (10x Genomics) according to the manufacturer's instructions. After barcoding, cDNA was amplified by PCR, and enzymatically hydrolyzed to form 200–300 bp fragments. Full‐length, barcoded cDNA was further amplified by PCR to generate sufficient mass for library construction. High‐throughput sequencing was performed on the constructed libraries using the double‐end sequencing mode of the Illumina sequencing platform. To obtain high‐quality cells, the low‐quality cells were removed based on the number of genes identified in a single cell, the total number of UMIs in a single cell, and the ratio of mitochondrial gene expression of UMIs in a single cell. Then, the Seurat package was used to normalize the data, and UMAP was used to visualize clustering and identify cell types. Pseudo‐time analysis was performed using the Monocle2 package, and the cells were arranged on a cell trajectory according to the pseudo‐time change to simulate the relationship of cell differentiation during the developmental process. Finally, CellPhoneDB was used to analyze the ligand‐receptor interactions between different single cells.^[^ ^75^ ^]^

### Cell Sorting Using Flow Cytometry

To obtain Lgr5^+^ stem cells, the *Lgr5* ^EGFP‐IRES‐creERT2^ mice were used to isolate single epithelial cells, and flow cytometry was performed to screen and collect the FITC‐positive population.

### Isolation and Culture of Intestinal Crypt Organoids

Isolation of intestinal crypt organoids was performed according to previously reported methods.^[^ ^76^ ^]^ Briefly, fresh small intestine tissues from WT and *Ddx58* ^−/−^ mice were cut longitudinally and washed with DPBS, then cut into small pieces of ≈2 mm. The tissues were transferred into a digestive solution (DPBS+1% P/S+2 mM EDTA) and shaken at 4 °C for 30 min. After vertical suspension for 15s, the supernatant was removed and the tissues were repeatedly cleaned with DPBS for 5–6 times, and then the tissues were transferred to the separation solution (DPBS+1% P/S+1% sorbitol + 1.5% sucrose). After vortex shaking and filtration through a 70 µm strainer, the filtrate was centrifuged at 200 g for 10 min at 4 °C. After resuspension and counting the crypt organoids, 800 organoids/well were embedded in Matrigel (Corning), seeded into 24‐well plates, and polymerized at 37 °C for 20 min. IntestiCult Organoid Growth Medium (Stem cell technologies) was added into plates and cultured at 37 °C with 5% CO_2_. After culturing for one day, the organoids received 4 Gy ionizing radiation, and images were captured at 24 and 48 h after irradiation. Organoids were collected, and RNA was extracted at 72 h after culture.

### Hematoxylin and Eosin Staining

The small intestine tissue was fixed in 4% paraformaldehyde immediately after isolation, and made into paraffin blocks after dehydration and embedding. The paraffin blocks were cut into 5 µm slices and placed on the slide. The slices were deparaffinized and then stained sequentially with hematoxylin and eosin. Finally, they were sealed with neutral gum. The stained tissues were observed under a microscope, and images were captured for analysis.

### Immunohistochemistry and Immunofluorescence

Paraffin sections of small intestinal tissues were deparaffinized, followed by antigen retrieval with Citrate (pH6.0) or EDTA (pH9.0). Once the slides were restored to room temperature, they were washed with PBS three times. The tissues were then blocked by hydrogen peroxide and 5% BSA, followed by incubation with primary antibodies at 4 °C overnight. The primary antibodies used were Olfm4 (Cell Signaling Technology, #39141), Ki67 (Abcam, ab15580), Lysozyme (Abcam, ab108508), GFP (Abcam, ab6556), Isg15 (F‐9) (Santa Cruz Biotechnology, sc‐166755), and PCNA (Proteintech, 60097‐1‐Ig). After washing off the primary antibodies with PBS, the slides were incubated with HRP‐linked secondary antibodies (ZSGB‐BIO) for immunohistochemistry staining, followed by staining with DAB and hematoxylin. The CY3‐conjugated goat anti‐mouse (Proteintech, SA00009‐1), CY3‐conjugated goat anti‐rabbit (Proteintech, SA00009‐2), FITC‐conjugated goat anti‐mouse (Proteintech, SA00003‐1), and FITC‐conjugated goat anti‐rabbit (Proteintech, SA00003‐2) secondary antibodies were used to perform immunofluorescence staining followed by sealing with DAPI (Vector Laboratories). Additionally, goblet cells were stained using the Alcian Blue‐Periodic Acid‐Schiff Staining Kit (Solarbio) according to the manufacturer's protocol.

### RNA Extraction and qRT‐PCR

Total RNA from Lgr5^+^ ISCs, crypts, and crypt organoids was extracted using TRIzol reagent (Thermo Fisher Scientific). Subsequently, cDNA was synthesized using PrimeScript RT reagent Kit (Takara), and real‐time PCR was performed using Evagreen 2x qPCR MasreMix‐No Dye (Applied Biological Materials) in accordance with the manufacturer's instructions. The expression of target genes was normalized to the housekeeping gene (*Gapdh*) and shown as 2^−ΔΔCt^. 16S gene copy numbers (total bacterial DNA) in feces, VLPs, FMT, and FVT components were detected using qPCR according to the previously reported methods.^[^ ^77^ ^]^ The primers used are listed in Table S1 (Supporting Information).

### Measurement of Inflammatory Cytokines

Plasma concentrations of inflammatory factors IL‐10, IL‐5, IL‐6 were measured by Milliplex Map Mouse Cytokine/Chemokine Panel 1 assay (Millipore) according to the manufacturer's protocol. All samples were tested simultaneously with quality control and standard samples provided in the kit using the Luminex 200 system.

### Statistical Analysis

Statistical analysis between two groups was performed using Student's *t‐*test, and the statistical analysis between multiple groups was performed using one‐way analysis of variance (one‐way ANOVA). Statistical analysis was completed using GraphPad Prism 8 software, and *P* < 0.05 indicated a significant difference between the two groups. (^*^ *P* < 0.05, ^**^ *P* < 0.01, ^***^ *P* < 0.001, ^****^ *P* < 0.0001).

### Ethics Approval

All animal studies were approved by the Animal Ethical and Welfare Committee of the Institute of Radiation Medicine of the Chinese Academy of Medical Sciences and Peking Union Medical College (IRM‐DWLL‐2019056).

## Conflict of Interest

The authors declare no conflict of interest.

## Author Contributions

X.Z. performed experiments and prepared the manuscript. Y. C., L. L., and Z. D. analyzed the sequencing data. Y.H., Y.W., T.W., X.L., J.M., and H.Z. performed experiments. Q.W., J.W., C.X., L.D., and S.F. investigate information. F.W., Q.L., and Y.L. reviewed and edited the manuscript. Y.L. designed the study and edited the manuscript.

## Supporting information



Supporting Information

## Data Availability

Sequence data that support the findings of this study have been deposited in the National Genomics Data Center and can be accessed through the project number PRJCA033983: CRA021572 (16S rRNA gene sequencing), CRA021562 (Metagenome of VLPs), CRA021557 (Single cell RNA sequence).
