# Supplementary material for: Commensal Viruses Promote Intestinal Stem Cell Regeneration Following Radiation Damage by Inhibiting Hyperactivation of RIG‐I and Notch Signals
Source: Adv Sci (Weinh). 2025 Jul 18;12(37):e05204. doi: 10.1002/advs.202505204 (PMC12499508; doi:10.1002/advs.202505204)
Supplement: Supplementary file 1 — Supporting Information [file ADVS-12-e05204-s001.docx]

**
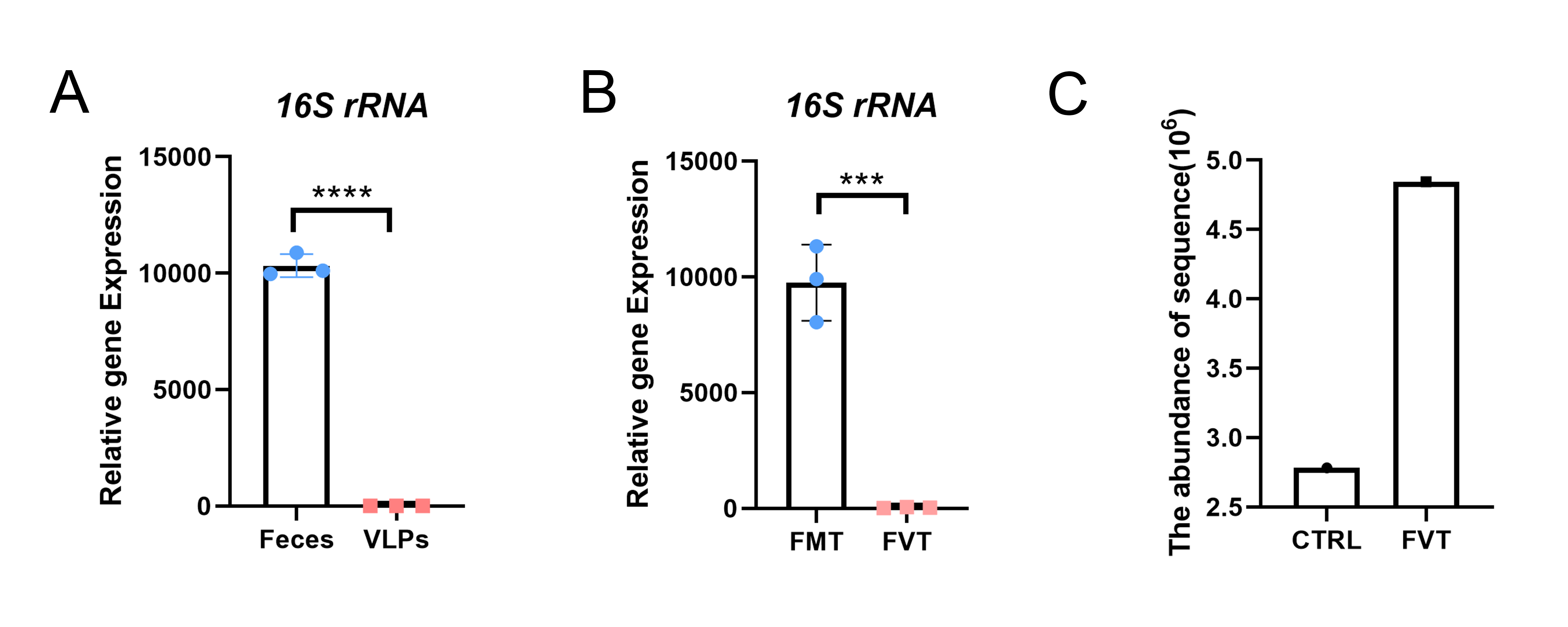
Figure S1**. Bacterial DNA and viral counts were measured.

1. The bacterial loading (bacterial DNA) was evaluated in feces and VLPs via 16S qPCR to ensure that there was no bacterial contamination in the VLPs.
2. The bacterial loading (16S rRNA) was evaluated in FMT and FVT components to ensure that there was no bacterial contamination in the FVT components.
3. The viral reads count number of sequencing results was analyzed in CTRL and FVT-treated mice to evaluate the enrichment of viruses by FVT.

**
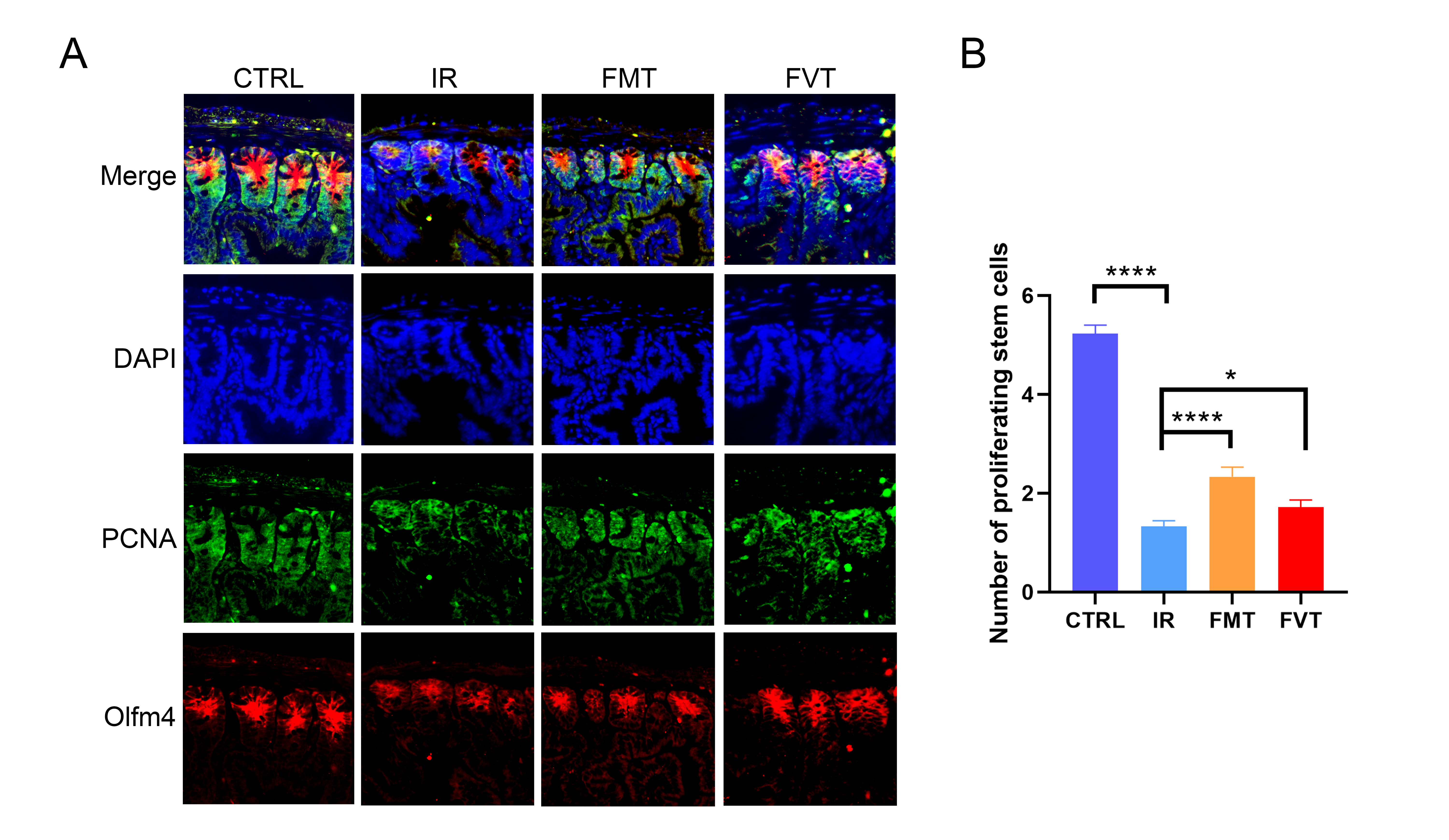
Figure S2**. FVT promoted the proliferation of stem cells after radiation damage.

1. Representative images of immunofluorescence staining of small intestinal tissue for PCNA and Olfm4 among different groups.
2. Quantitative analysis of the number of cells co-expressing PCNA and Olfm4.

Data presented as mean ± SEM. **P* < 0.05; ***P* < 0.01; ****P* < 0.001; *****P*< 0.0001.

**

Figure S3**. FVT alleviated radiation-induced hematopoietic damage.

1. Peripheral blood analysis of mice was measured at day 7 after 18 Gy irradiation in different group. The number of white blood cell (WBC) among different groups.
2. The number of lymphocyte count (LY) among different groups.
3. The percentage of lymphocytes (LY%) among different groups.
4. The percentage of monocytes (MO%) among different groups.

Each dot represented one mouse (n=4-6). Data represent the mean ± SEM, **P* < 0.05, ***P* < 0.01, ****P* < 0.001, *****P* < 0.0001.

**
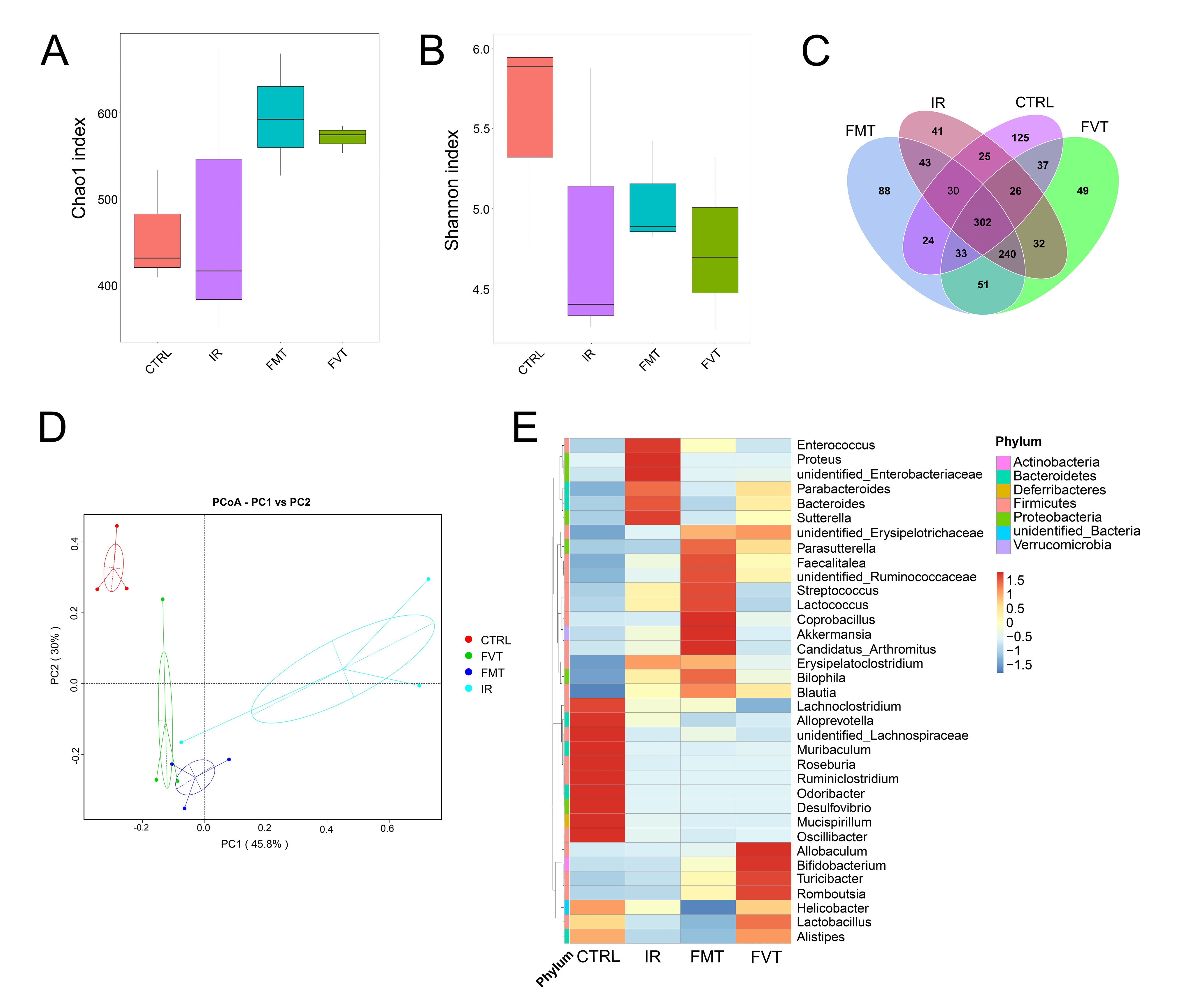
Figure S4**. Diversity and abundance of bacterial communities following healthy mice fecal virome administration.

(A-B) Bacterial diversity was determined by Chao1 and Shannon index after IR, FMT, and FVT treatment.

(C) Venn diagrams revealed the different OTU numbers among four groups. Each group contained three independent samples.

(D) β diversity of bacteria was evaluated by Principal Coordinate Analysis (PCoA) on weighted UniFrac distance among CTRL, IR, FMT, and FVT groups.

(E) Heatmap showed the relative abundance at the genus level of bacteria for CTRL, IR, FMT, and FVT groups.

**
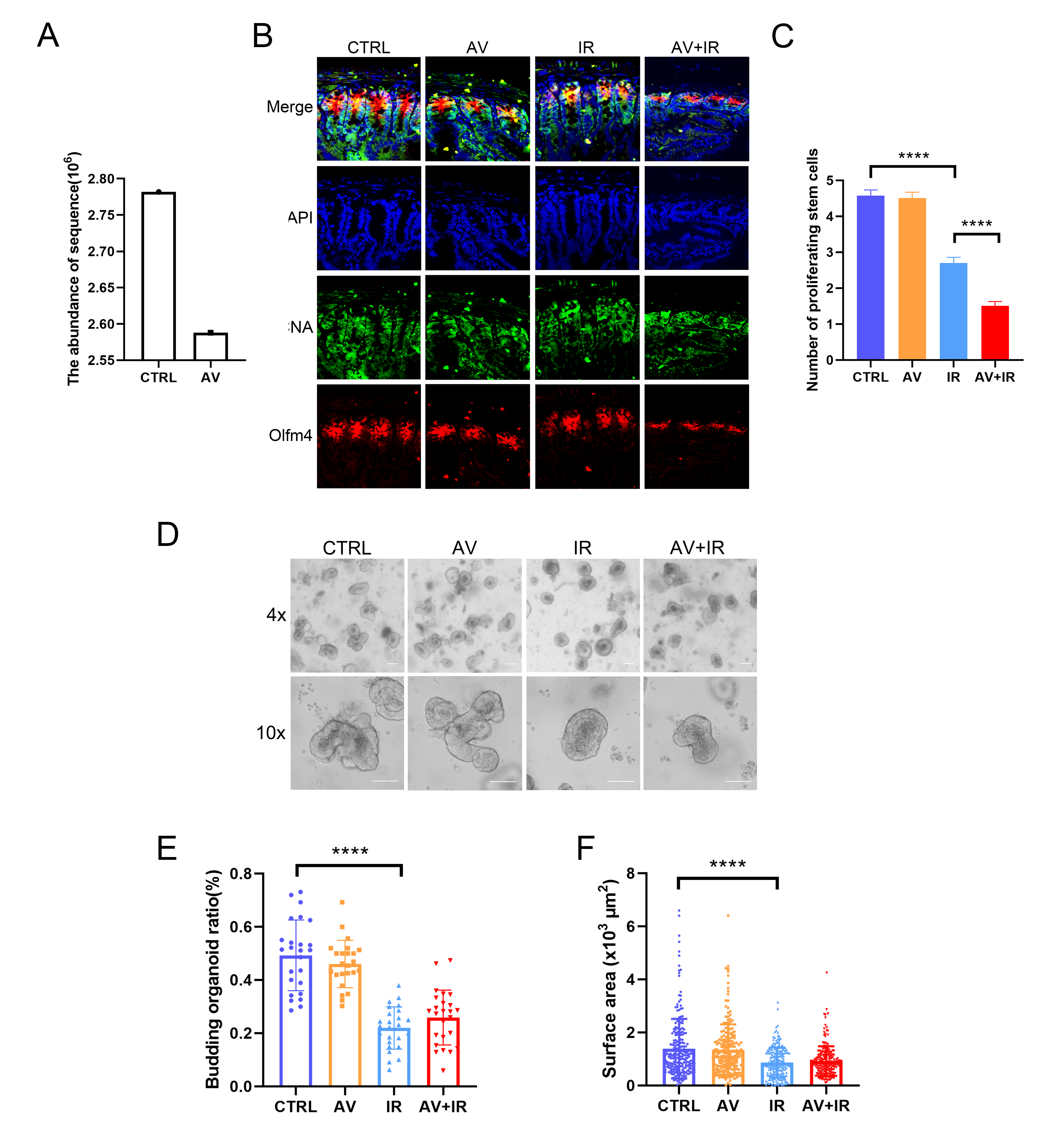
Figure S5**. Antiviral cocktail decreased the proliferation of stem cells in irradiated mice but did not impair organoid growth after radiation in the absence of the virus.

1. The viral reads count number of sequencing results of mice in control and AV group.
2. Representative images of the immunofluorescence staining of small intestinal tissue for PCNA and Olfm4 among different groups.
3. Quantitative analysis of the number of cells co-expressing PCNA and Olfm4.

(D-F) Representative images and quantitative analysis of organoid formation after AV treatment and at 24h post-radiation. Data presented as mean ± SEM. **P* < 0.05; ***P* < 0.01; ****P* < 0.001; *****P* < 0.0001.

**
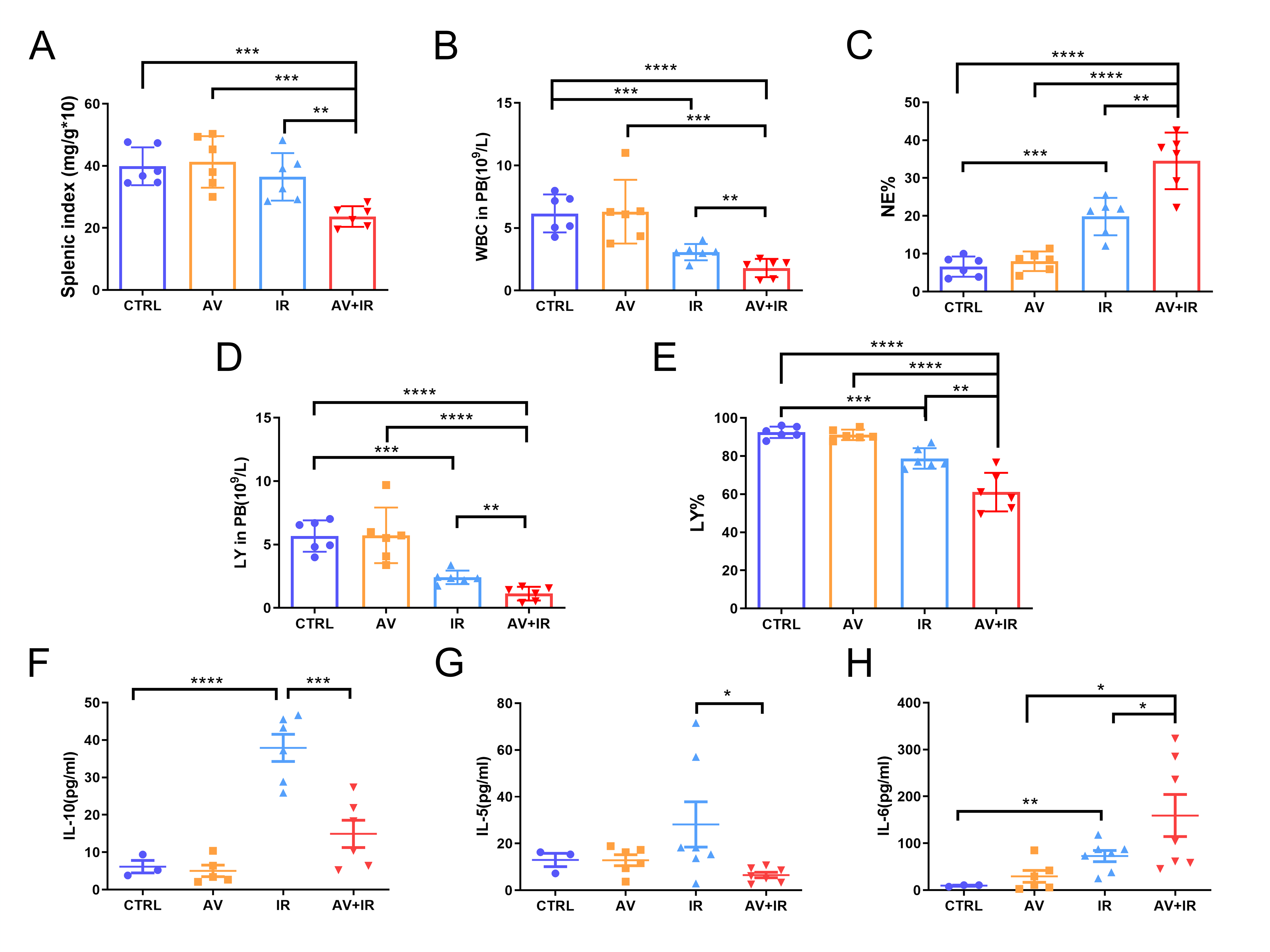
Figure S6**. Antiviral cocktail aggravated radiation-induced hematopoietic injury.

1. Spleen index 5 days after radiation (n=6).

(B-E) Peripheral blood analysis of mice in different group. White blood cell count (WBC) (B), the percentage of neutrophils (NE%) (C), lymphocyte count (LY) (D), the percentage of lymphocytes (LY%) (E), were measured at day 5 after 15 Gy radiation (n=6).

(F-H) Anti-inflammatory IL-10 (F) and IL-5 (G), or pro-inflammatory cytokines IL-6 (H) were detected in plasma 5 days after irradiation (n=3 in CTRL group and n=6 in other three groups).

Each dot represented one mouse. Data presented as mean ± SEM. **P* < 0.05; ***P* < 0.01; ****P* < 0.001; *****P* < 0.0001.

**Figure S7**. Single-cell sequencing revealed heterogeneity of intestinal epithelial cells.

1. UMAP plot of the 38165 epithelial cells revealed 25 cellular clusters.

(B) Annotation results for different cell types.

(C) Pseudo-time analysis showed three trajectories including seven types of epithelial cells.

(D) Heatmap of differential gene expression in eight stem cell subclusters.

(E) GO functional enrichment analysis of upregulated genes in ISG Lgr5^lo^ ISCs cluster.

(F) The transcriptional levels of proliferating genes in ISG Lgr5^lo^ ISCs cluster.

**
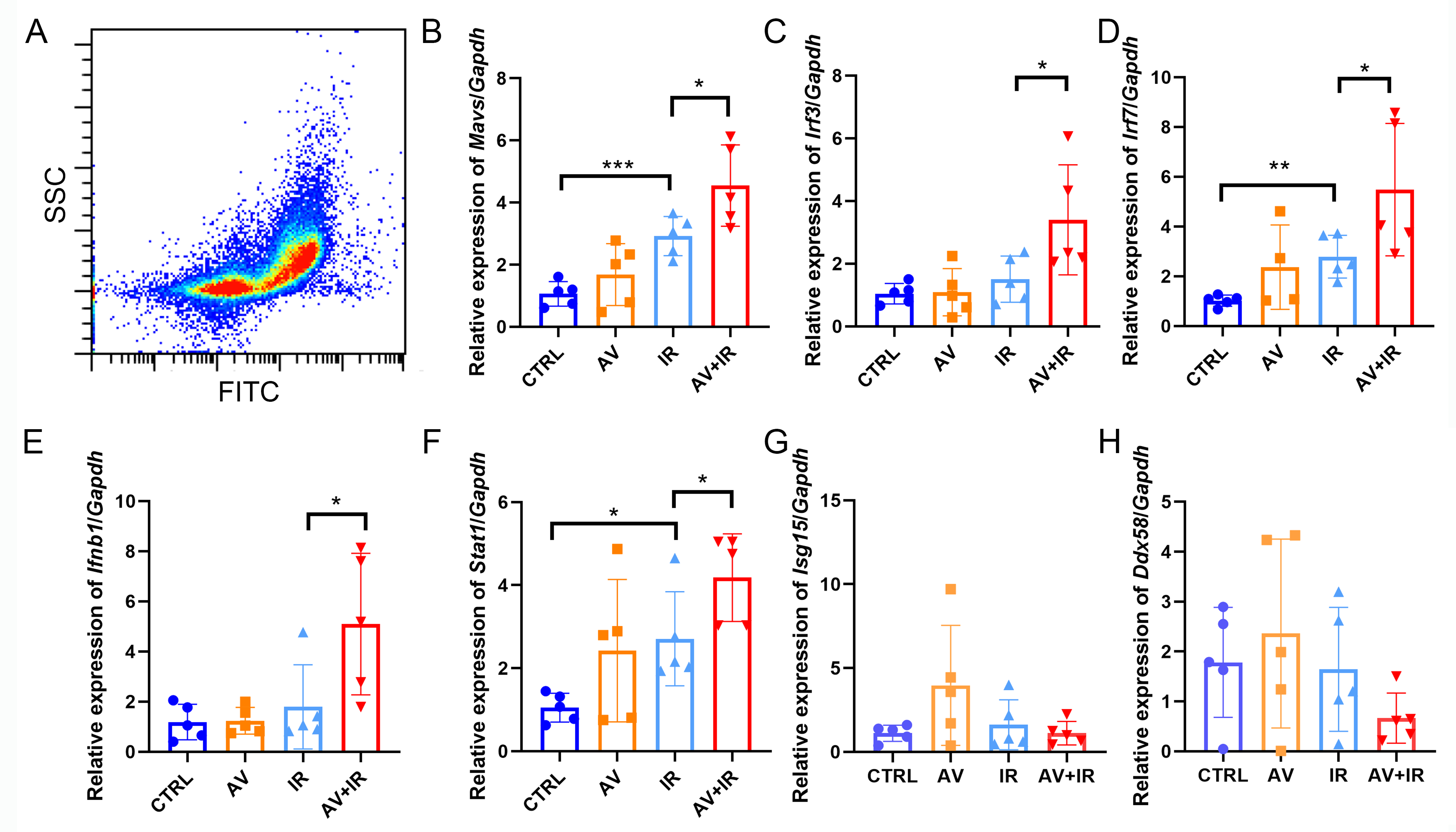
Figure S8**. Decreased viral load activated RIG-I-like receptor signal in Lgr5^+^ stem cells without effecting *Isg15* and *Ddx58* expression in intestine tissues.

1. Representative image of flow sorting of *Lgr5*^EGFP-IRES-CreERT2^ mice intestinal epithelial cells.

(B-F) The gene expression of RIG-I-like receptor signaling pathway in Lgr5^+^ stem cells after AV treatment and radiation.

(G) qPCR analysis of *Isg15* expression in intestine tissues.

(H) qPCR analysis of *Ddx58* expression in intestine tissues.

Each dot represented one mouse (n=5). Data presented as mean ± SEM. **P* < 0.05; ***P* < 0.01; ****P* < 0.001; *****P*< 0.0001.

**Figure S9**. AV treatment decreased the number of proliferating of stem cells in irradiated wild-type
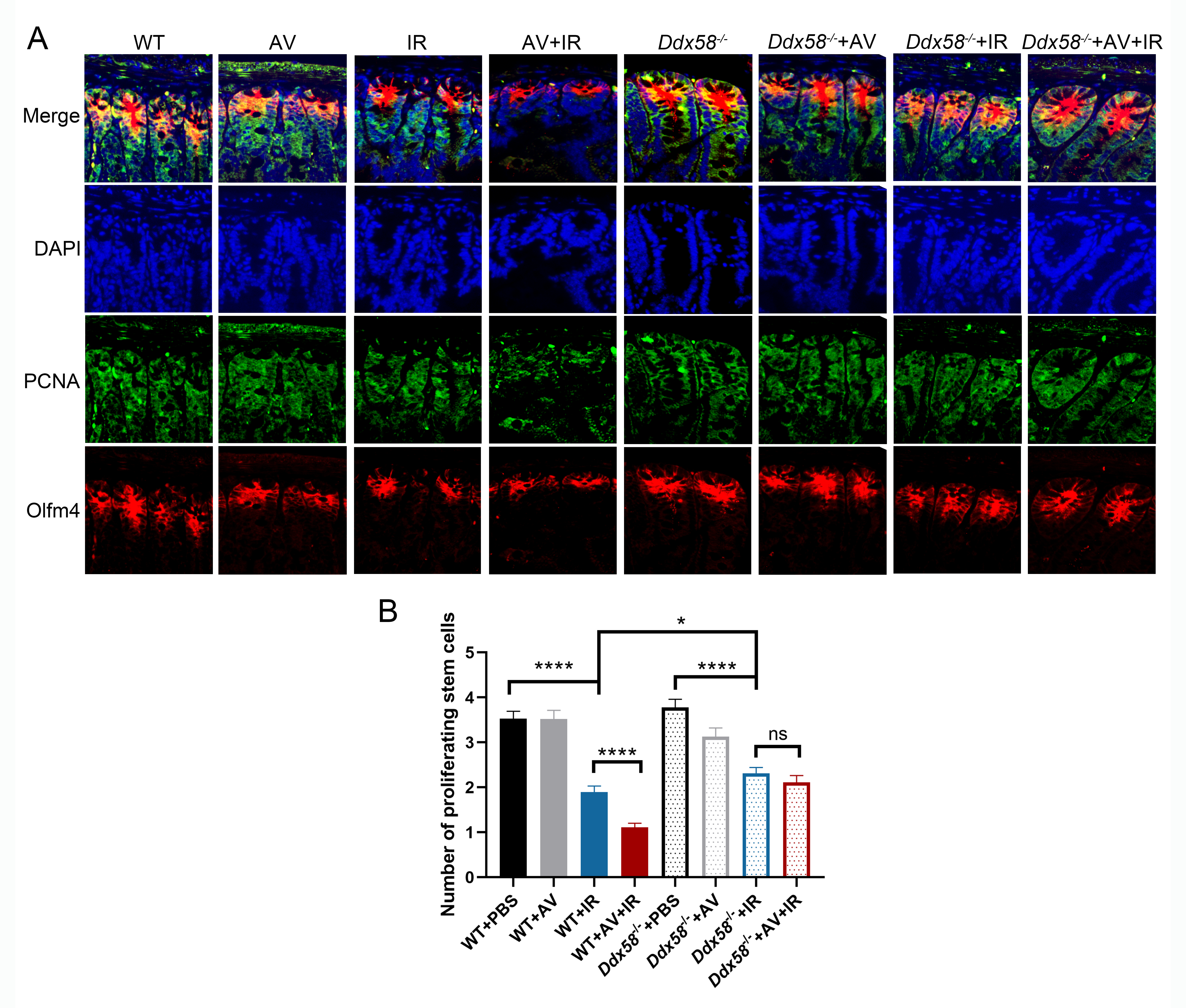
mice but had no effect in irradiated *Ddx58^-/-^* mice.

1. Representative images of immunofluorescence staining of small intestinal tissue for PCNA and Olfm4 among different groups.
2. Quantitative analysis of the number of cells co-expressing PCNA and Olfm4.

Data presented as mean ± SEM. **P* < 0.05; ***P* < 0.01; ****P* < 0.001; *****P*< 0.0001.

**Figure S10**. Antiviral cocktail did not exacerbate the radiation-induced hematopoietic injury in
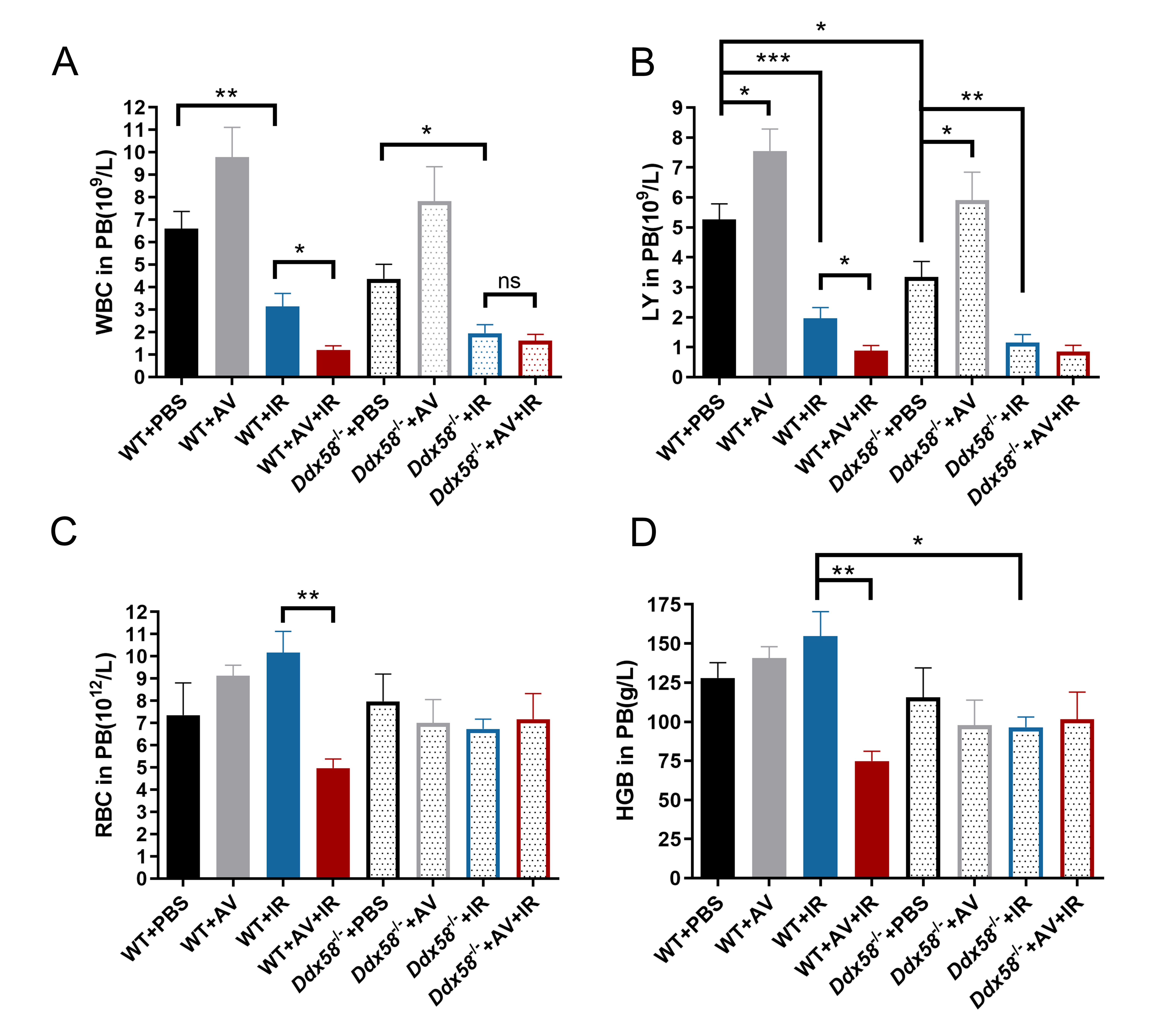
*Ddx58^-/-^* mice.

1. Peripheral blood analysis of mice was measured after 15 Gy irradiation. The number of white blood cell (WBC) among different groups.
2. The number of lymphocyte count (LY) among different groups.
3. The number of red blood cell (RBC) among different groups.
4. The level of hemoglobin (HGB) among different groups.

Data presented as mean ± SEM. **P* < 0.05; ***P* < 0.01; ****P* < 0.001; *****P* < 0.0001.


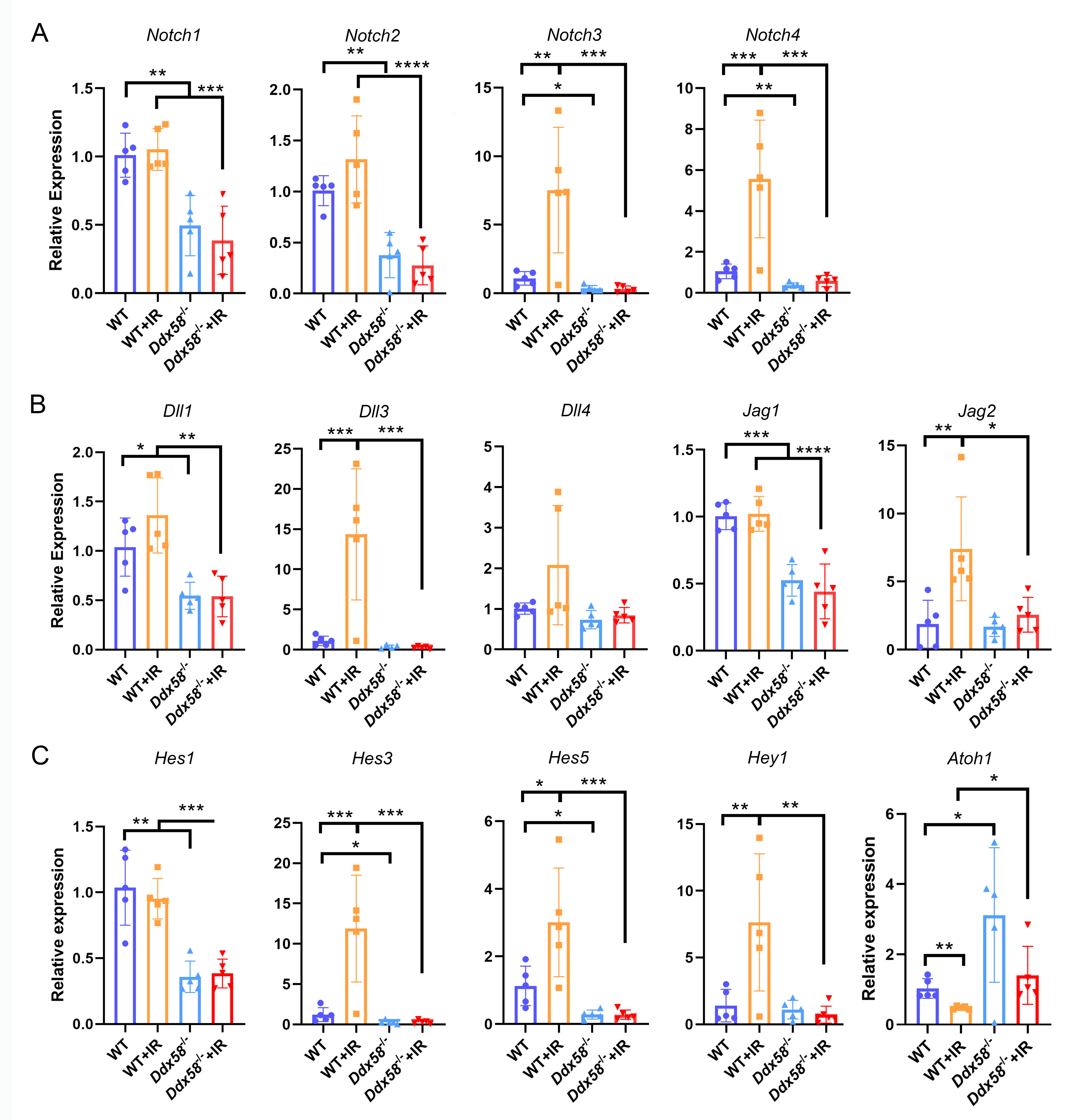
**Figure S11**. RIG-I deficiency inhibited Notch signaling in the crypts of mice.

(A) The expression of Notch receptors in the crypts of wild-type and RIG-I deficiency mice with or without radiation.

(B) The expression of Notch ligands in the crypts of wild-type and RIG-I deficiency mice with or without radiation.

(C) The expression of Notch target genes and *Atoh1* in the crypts of wild-type and RIG-I deficiency mice with or without radiation. Each dot represented one mouse. Data presented as mean ± SEM. **P* < 0.05; ***P* < 0.01; ****P* < 0.001; *****P*< 0.0001.

**Table S1** Primers used in this study.

| *Notch1* | forward | CCCTTGCTCTGCCTAACGC |
| --- | --- | --- |
|  | reverse | GGAGTCCTGGCATCGTTGG |
| *Notch2* | forward | GACTGCCAATACTCCACCTCT |
|  | reverse | CCATTTTCGCAGGGATGAGAT |
| *Notch3* | forward | AGTGCCGATCTGGTACAACTT |
|  | reverse | CACTACGGGGTTCTCACACA |
| *Notch4* | forward | GAACGCGACATCAACGAGTG |
|  | reverse | GGAACCCAAGGTGTTATGGCA |
| *Dll1* | forward | CCCATCCGATTCCCCTTCG |
|  | reverse | GGTTTTCTGTTGCGAGGTCATC |
| *Dll3* | forward | CTGGTGTCTTCGAGCTACAAAT |
|  | reverse | TGCTCCGTATAGACCGGGAC |
| *Dll4* | forward | TTCCAGGCAACCTTCTCCGA |
|  | reverse | ACTGCCGCTATTCTTGTCCC |
| *Jag1* | forward | AATCGCATCGTACTGCCTTTC |
|  | reverse | GTGTCATTACTGGAATCCCAGG |
| *Jag2* | forward | TTCTGTGACGAGTGTGTCCC |
|  | reverse | GCGCAGAGGTATTGGTCAGG |
| *Hes1* | forward | GCAGCTTGGCTGTGGTAGA |
|  | reverse | TGCTGAGCGCGGCTTCTAT |
| *Hes3* | forward | GCACGCATCAACGTGTCAC |
|  | reverse | CCAGCTTTCGTTTCCGTATCTG |
| *Hes5* | forward | AGTCCCAAGGAGAAAAACCGA |
|  | reverse | GCTGTGTTTCAGGTAGCTGAC |
| *Hey1* | forward | ACTGAGCGTGAGTGGGATCA |
|  | reverse | CGCCGAACTCAAGTTTCCATT |
| *Atoh1* | forward | GGGAAGCCCCGTGACAAATA |
|  | reverse | TGGACCATGAAACGATGCCA |
| *Gapdh* | forward | AGGTCGGTGTGAACGGATTTG |
|  | reverse | TGTAGACCATGTAGTTGAGGTCA |
| *Ddx58* | forward | AAGAGCCAGAGTGTCAGAATCT |
|  | reverse | AAGAGCCAGAGTGTCAGAATCT |
| *Isg15* | forward | GGTGTCCGTGACTAACTCCAT |
|  | reverse | TGGAAAGGGTAAGACCGTCCT |
| *Mavs* | forward | CTGCCAACACAATACCACCTGAG |
|  | reverse | TCTCTGGTCCAGAGTGCAAGCT |
| *Irf3* | forward | GAGAGCCGAACGAGGTTCAG |
|  | reverse | CTTCCAGGTTGACACGTCCG |
| *Irf7* | forward | CACCCCCATCTTCGACTTCA |
|  | reverse | CCAAAACCCAGGTAGATGGTGTA |
| *Ifnb1* | forward | CGTGGGAGATGTCCTCAACT |
|  | reverse | AGATCTCTGCTCGGACCACC |
| *Stat1* | forward | TCACAGTGGTTCGAGCTTCAG |
|  | reverse | GCAAACGAGACATCATAGGCA |
| 16S | forward | ACTCCTACGGGAGGCAGCAGT |
|  | reverse | ATTACCGCGGCTGCTGGC |
